# Season‐modulated responses of Neotropical bats to forest fragmentation

**DOI:** 10.1002/ece3.3005

**Published:** 2017-05-13

**Authors:** Diogo F. Ferreira, Ricardo Rocha, Adrià López‐Baucells, Fábio Z. Farneda, João M. B. Carreiras, Jorge M. Palmeirim, Christoph F. J. Meyer

**Affiliations:** ^1^ Centre for Ecology, Evolution and Environmental Changes (cE3c)Faculty of SciencesUniversity of LisbonLisbonPortugal; ^2^ Biological Dynamics of Forest Fragments ProjectNational Institute for Amazonian Research and Smithsonian Tropical Research InstituteManausBrazil; ^3^ Metapopulation Research CentreFaculty of BiosciencesUniversity of HelsinkiHelsinkiFinland; ^4^ Faculty of Life SciencesUniversity of MadeiraFunchalPortugal; ^5^ Ecosystems and Environment Research Centre (EERC)School of Environment and Life SciencesUniversity of SalfordSalfordUK; ^6^ Museu de Ciències Naturals de GranollersGranollersCatalunyaSpain; ^7^ Department of Ecology/PPGEFederal University of Rio de JaneiroRio de JaneiroBrazil; ^8^ National Centre for Earth Observation (NCEO)University of SheffieldSheffieldUK

**Keywords:** Chiroptera, fragmentation, landscape structure, local vegetation structure, seasonality

## Abstract

Seasonality causes fluctuations in resource availability, affecting the presence and abundance of animal species. The impacts of these oscillations on wildlife populations can be exacerbated by habitat fragmentation. We assessed differences in bat species abundance between the wet and dry season in a fragmented landscape in the Central Amazon characterized by primary forest fragments embedded in a secondary forest matrix. We also evaluated whether the relative importance of local vegetation structure versus landscape characteristics (composition and configuration) in shaping bat abundance patterns varied between seasons. Our working hypotheses were that abundance responses are species as well as season specific, and that in the wet season, local vegetation structure is a stronger determinant of bat abundance than landscape‐scale attributes. Generalized linear mixed‐effects models in combination with hierarchical partitioning revealed that relationships between species abundances and local vegetation structure and landscape characteristics were both season specific and scale dependent. Overall, landscape characteristics were more important than local vegetation characteristics, suggesting that landscape structure is likely to play an even more important role in landscapes with higher fragment‐matrix contrast. Responses varied between frugivores and animalivores. In the dry season, frugivores responded more to compositional metrics, whereas during the wet season, local and configurational metrics were more important. Animalivores showed similar patterns in both seasons, responding to the same group of metrics in both seasons. Differences in responses likely reflect seasonal differences in the phenology of flowering and fruiting between primary and secondary forests, which affected the foraging behavior and habitat use of bats. Management actions should encompass multiscale approaches to account for the idiosyncratic responses of species to seasonal variation in resource abundance and consequently to local and landscape scale attributes.

## 1 Introduction

Throughout the tropics, high rates of deforestation have drastically increased the number of old‐growth forest patches surrounded by an anthropogenically modified matrix (Melo, Arroyo‐Rodríguez, Fahrig, Martínez‐Ramos, & Tabarelli, 2013). These modified matrices can act as a hostile environment and as a selective filter to wildlife, influencing the connectivity between remnant forest patches (Gascon et al., 1999). However, recent research has demonstrated that some anthropogenically modified habitats are not completely inhospitable and can be crucial for the survival of numerous animal species in today's expanding tropical agricultural landscapes (Kupfer, Malanson, & Franklin, 2006; Mendenhall, Karp, Meyer, Hadly, & Daily, 2014; Williams‐Guillén, Olimpi, Maas, Taylor, & Arlettaz, 2016). Forest regrowth on abandoned agricultural lands and logged areas has led to the expansion of secondary forests throughout the tropics, representing one‐sixth of all primary forest that was cut during the 1990s (Chazdon et al., 2009; Wright, 2005). These secondary forest matrices are structurally more similar to primary forest than other types of anthropogenic matrices such as agricultural fields (Ferreira & Prance, 1999). Consequently, recent research has highlighted their importance in terms of resources for foraging, nesting, and protection for an array of animal taxa (Chazdon et al., 2009) and as corridors that can help to mitigate the impacts of deforestation (Bobrowiec & Gribel, 2010).

In the tropics, seasonality is marked not by a difference in temperature but by a difference in precipitation (MacArthur, 1984). Differences in precipitation between seasons affect plant production, causing oscillations in resource availability, which in turn affects the presence and abundance of animal species (Avila‐Cabadilla et al., 2014; Beja et al., 2009; Castro & Espinosa, 2015; Ramos Pereira, Marques, & Palmeirim, 2010). In fragmented landscapes, natural fluctuations in resource availability can be altered at forest edges (Ewers & Banks‐Leite, 2013) and in the human‐modified matrix (Chazdon et al., 2009) as a result of different microclimatic conditions. Furthermore, fragmentation can disrupt seasonal movements and hinder access to key resources (Kattan, Alvarez‐Lopez, & Giraldo, 1994). For instance, during seasons of low food availability, tropical vertebrates such as many bird species may make greater use of small fragments to expand their foraging areas or use them as stepping stones to disperse to areas of higher food availability (Maldonado‐Coelho & Marini, 2004). Hence, seasonality can exacerbate the impacts of fragmentation, especially for species that are not able to overcome the matrix's ecological barriers to exploit available resources in other areas.

Bats play an important role in the maintenance of tropical ecosystems through seed dispersal, pollination, and regulation of invertebrate populations (Kunz, Braun de Torrez, Bauer, Lobova, & Fleming, 2011). However, like many other groups of wildlife, bats are affected by deforestation and habitat degradation, and over the years, numerous studies have documented the variability in responses of neotropical bats to these perturbations (reviewed in Meyer, Struebig, & Willig, 2016).

Many studies have shown that responses to habitat fragmentation at the assemblage level are often hard to detect, but that there are often marked responses at the population level (Meyer et al., 2016). Responses at the population level are highly species and ensemble specific (Avila‐Cabadilla, 2012; Chambers, Cushman, Medina‐Fitoria, Martínez‐Fonseca, & Chávez‐Velásquez, 2016; Galitsky & Lawler, 2015; Klingbeil & Willig, 2009; Moura et al., 2016), highlighting the need for studies to focus on the level of individual species. Although many studies across the neotropics have assessed the impacts of fragmentation on bats at the population and assemblage level, few were conducted over longer periods and consequently seasonal variation in species responses were rarely considered (Meyer et al., 2016). However, Cisneros, Fagan, and Willig (2015a) and Klingbeil and Willig (2010) found that phyllostomid bats had divergent responses to landscape structure between seasons, whereby some ensembles/species responded to landscape composition (e.g., forest cover) in one season and to landscape configuration (e.g., edge density) in the other season.

Despite the importance of landscape context for ecological processes, it has been shown, for different taxa, that landscape structure can have a less important role in determining ecological patterns than local habitat metrics (Collinge, 2009). In this context, responses to landscape metrics and local vegetation structure are often species and ensemble specific (e.g., Galitsky & Lawler, 2015; Lee & Carroll, 2014). For example, the activity of temperate forest‐dwelling bats may be better predicted by local vegetation structure than by landscape‐level attributes (Erickson & West, 2003). Responses of tropical bats to fragmentation at the landscape level are likely modulated by local‐scale vegetation structure and influenced by season‐specific variation in biotic and abiotic conditions, highlighting the importance of integrated approaches. Nevertheless, studies that jointly explore the interactive effects of seasonality and local as well as landscape variables on bat population‐ and ensemble‐level responses in tropical fragmented landscapes are so far lacking.

In this study, we assessed how general patterns of bat abundance changed between the wet and dry seasons in primary forest fragments, continuous forest controls, and in the secondary forest matrix in a tropical fragmented landscape. In addition, we analyzed the influence of vegetation structure (local‐scale variable) and, for five spatial scales, metrics of landscape composition and configuration on the abundance of eight bat species and evaluated whether the relative importance of local, compositional, or configurational characteristics varied between dry and wet seasons. As per the findings of Klingbeil and Willig (2010), we expected bat responses to landscape structure to be season and species specific, and that different ensembles (animalivores and frugivores) would respond differently to seasonality. Specifically, we anticipated that frugivorous species would respond more to compositional metrics in the dry season and to configurational metrics in the wet season. These responses would reflect the higher fruit availability in secondary forest during the dry season and the diversity of food sources available across the primary forest during the wet season. We also anticipated that animalivorous species would respond mostly to landscape composition in the wet season due to higher insect availability and more to configurational metrics in the dry season due to the increasing need of bats to visit habitats of lower quality (i.e., matrix or edge) to meet their dietary needs. Further, we anticipated local vegetation structure to play a greater role than landscape structure in the wet season, due to higher food availability and smaller home ranges of bats during this period (Haugaasen & Peres, 2005; Klingbeil & Willig, 2010). These patterns would reflect the reproductive cycle of bat species, the availability, and distribution of food resources across the landscape and the differential ability of species to exploit the resources in the secondary forest matrix. Finally, we predicted that abundances of frugivores in secondary forest would be higher than those of animalivores, more specifically, that abundances will be more similar to those in continuous forest with increasing successional stage of secondary forest, following the gradient of increasing similarity in vegetation structure and composition.

## 2 Methods

### 2.1 Study area

This study was carried out at the Biological Dynamics of Forest Fragments Project (BDFFP), located about 80 km north of Manaus, Central Amazon, Brazil. The climate of the region is classified as Am in the system of Köppen (Mesquita, Ickes, Ganade, & Bruce Williamson, 2001), with a mean annual temperature of 26.7°C (Haugaasen & Peres, 2005). There are two well‐defined seasons: A dry season from July to November when precipitation drops below 100 mm/month and a wet season from November to June when precipitation can exceed 300 mm/month. The type of forest present at the BDFFP is *terra firme* forest. Flowering and fruiting peaks occur in the dry season and in the beginning of the wet season, respectively (Haugaasen & Peres, 2005).

Between 1980 and 1984, eleven fragments were experimentally isolated in undisturbed continuous forest: five 1 ha fragments, four 10 ha fragments, and two 100 ha fragments. The fragments were initially surrounded by a matrix of cattle pasture. However, due to land use abandonment, a matrix of secondary forest has developed since then. The matrix now consists of secondary forest in different successional stages (Carreiras, Jones, Lucas, & Gabriel, 2014), dominated by *Vismia* spp. in areas that were cleared and burned, and by *Cecropia* spp. in areas that were cleared without fire (Mesquita, Massoca, Jakovac, Bentos, & Williamson, 2015).

For a more detailed description of the study area and experimental manipulation, see Laurance et al. (2011).

### 2.2 Experimental design

**Figure 1:**
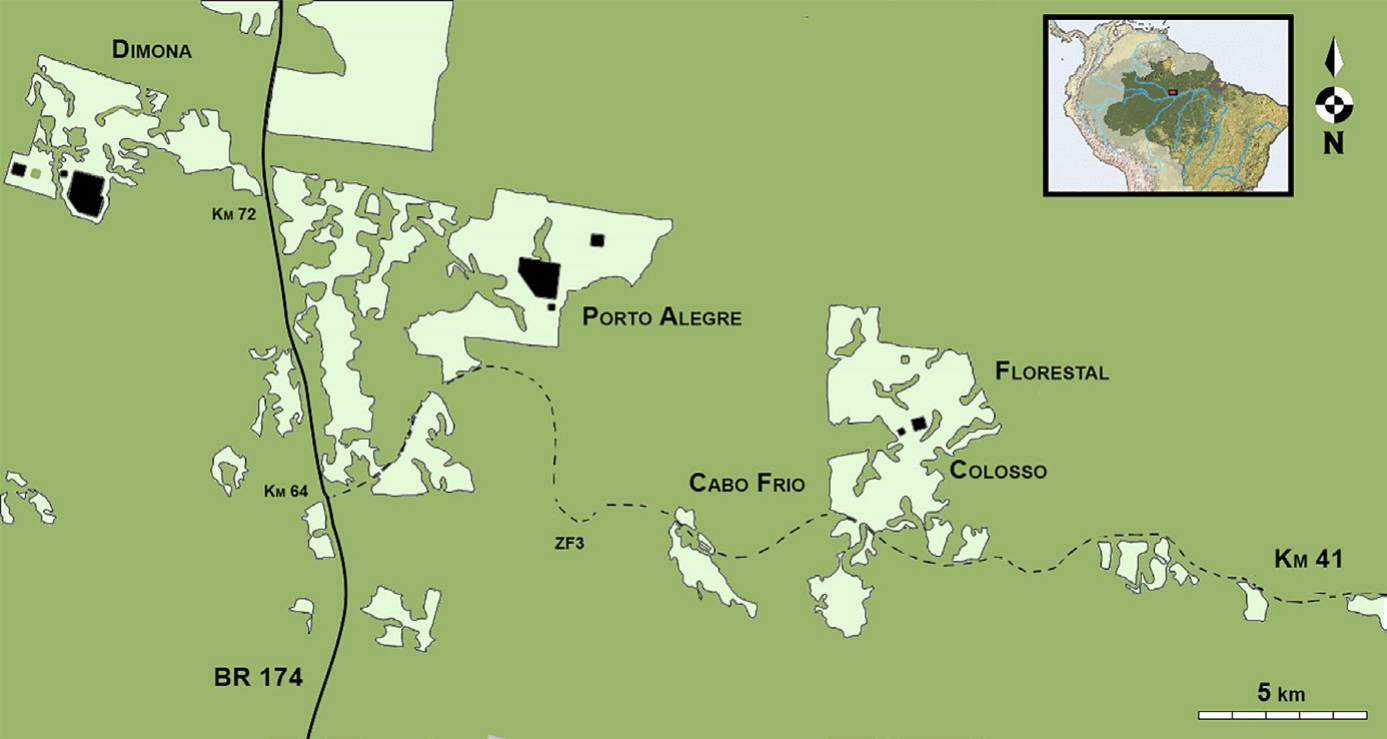
Map of the Biological Dynamics of Forest Fragments Project (BDFFP) study area in the Central Amazon. Black: sampling sites in forest fragments and continuous forest reserves. See Figure S2 for a detailed distribution of the 39 sampling points; light green: secondary forest matrix; dark green: continuous primary forest

The bat fauna was sampled in eight primary forest fragments—three of 1 ha, three of 10 ha, and two of 100 ha (Dimona, Porto Alegre and Colosso reserves)—and nine control sites spread over three areas of continuous primary forest (Cabo Frio, Florestal and Km41 reserves; Figure 1). Each fragment was sampled in the interior, at the edge, and in the adjacent matrix of secondary forest. Fragment interior sites were located on average 245 ± 208 m (mean ± SD) away from the fragment edge. Adjacent matrix sites were sampled 100 m from each fragment border. In continuous forest, a similar experimental design was used, with nine interior sites (three in each reserve), three edge sites, and three adjacent matrix sites. Mean distances between interior and edge sites in continuous forest were 1118 ± 488 m. Hence, a total of 39 sites were sampled (17 interior sites, 11 edge sites, and 11 matrix sites).

### 2.3 Bat sampling

Bats were captured using ground‐level mist nets during the dry season, between July and November of 2011 and 2012, and the wet season, between February to June of 2012 and 2013. Each interior site was surveyed eight times, four times in each season. The number of visits to edge and matrix sites ranged from 3 to 6 in the wet season and 2 to 3 in the dry season. For each survey, 14 mist nets (12 × 2.5 m, 16 mm mesh, ECOTONE, Poland) were used in continuous forest and fragment interiors, and seven mist nets at the edge and adjacent matrix sites. Nets were left open during 6 hr from dusk to midnight and were visited at intervals of ~20 min. The same site was never surveyed during two consecutive nights to avoid net shyness‐related capture bias (Marques et al., 2013). Adult bats (excluding pregnant females) were marked with numbered ball‐chain necklaces (*Pteronotus parnellii* and frugivores) or transponders (gleaning animalivores). Species identification followed Gardner (2008) and Lim and Engstrom (2001), and taxonomy follows Gardner (2008). The analyses were limited to phyllostomids and *P. parnellii* due to under‐representation of other families and species with this type of sampling method (Kalko, 1998).

### 2.4 Environmental characteristics

#### 2.4.1 Local vegetation structure

For each of the 39 sites, we quantified nine vegetation characteristics (canopy cover, canopy height, average of the DBH measures of trees ≥10 cm, vertical foliage density and number of lianas, palms, woody stems, trees and *Vismia* and *Cecropia* trees) within three 100 m^2^ (5 × 20 m) plots established 5 m from each side of the mist net transects. Values for each sampling site were calculated as the average across replicated plots. See Rocha et al. (2017) for a detailed description of the methodology used.

All vegetation variables were log(*x* + 1) transformed to reduce skewness. To reduce the dimensionality of the data, we performed a principal components analysis (PCA). Prior to the analysis, a *z*‐score standardization was carried out, that is, variables were standardized to a mean of zero and a standard deviation of one. The first axis explained 42.02% of the total variance and was positively associated with the average diameter at breast height of trees ≥10 cm, canopy height, canopy cover, number of palms and trees and vertical foliage density, and negatively associated with number of woody stems, lianas, and *Vismia* and *Cecropia* trees (Figure S1; Table S1). The scores of the first axis (PCA1) were used as predictor variable summarizing local vegetation structure.

#### 2.4.2 Landscape structure

Measurements of landscape characteristics were obtained using a 30‐m spatial resolution land cover map of the BDFFP landscape from 2011. This map was based on the analysis of an extensive (quasi‐annual) time series of Landsat Thematic Mapper data acquired since the inception of deforestation in the region (1970s) and up to 2011 (Carreiras et al., 2014). For the purpose of this study, the map was classified into four land cover types, representing continuous primary forest as well as different successional stages of the secondary forest matrix (initial: ≤5 years, intermediate: 6–15 years, advanced: ≥16 years) (see Carreiras et al., 2014; Figure S2). To assess scale dependency in bat responses to fragmentation, we used buffers of five different sizes (250, 500, 750, 1,000, 1,500 m radii) centered on each of the 39 sampling sites. These focal scales were selected in order to encompass the home ranges of different‐sized bat species (Meyer & Kalko, 2008a) and to avoid overlap between buffers and thus spatial autocorrelation. As performed elsewhere (Arroyo‐Rodríguez, Rojas, Saldaña‐Vázquez, & Stoner, 2016; Cisneros, Fagan, & Willig, 2015a; Cisneros, Fagan, & Willig, 2015b; Klingbeil & Willig, 2009, 2010), landscape structure was characterized by compositional and configurational landscape metrics, the former representing the proportions of the different habitat types in the landscape and the latter the spatial arrangement of habitat patches and connectivity between them (McGarigal & McComb, 1995). For each of the five focal scales, we calculated four compositional metrics: primary forest cover (PFC), secondary forest cover—initial stage (SFC1), intermediate stage (SFC2) and advanced stage (SFC3). In addition, we calculated four configurational metrics: edge density (ED), patch density (PD), mean nearest‐neighbour distance (MNND), and mean shape index (MSI). Landscape metrics were selected based on previous fragmentation studies on bats (Cisneros, Fagan, & Willig, 2015a; Cisneros, Fagan, & Willig, 2015b; Klingbeil & Willig, 2009, 2010; Meyer & Kalko, 2008a; Rocha et al., 2017). All metrics were calculated using the R package “SDMtools” (VanDerWal et al., 2015) except MNND, which was calculated using the software QGIS. This metric corresponds to the mean of the shortest straight‐line distance between the focal patch (sampling site) and each of its nearest neighbor of the same class (McGarigal, 2014). When a given buffer contained only one patch of primary forest, we calculated MNND as the distance between that patch and the nearest one in the next larger buffer.

### 2.5 Data analysis

#### 2.5.1 Influence of season and habitat type on bat abundance patterns

General linear mixed‐effects models (GLMMs) were used to assess differences in the abundance of species between seasons (dry and wet) and habitat types (interior, edge and matrix). All models were fitted using the glmer function in the “lme4” package in R (Bates, 2010). The abundance of a given species (number of individuals captured per species) was used as dependent variable (Poisson's distribution, log‐link function) and season and habitat type as predictors, implemented as an interaction effect. Models incorporated a random term accounting for the nested sampling design (i.e., site within BDFFP's reserves) and an offset with a site's total capture effort (log number of mist net hours; 1 mist net hour [mnh] equals one 12‐m net open for 1 hr). For each species, significance of the predictors was assessed with likelihood‐ratio tests, and significant results were analyzed further via multiple comparison tests with Tukey's contrasts (adjusted *p*‐values reported) using the R package “*multcomp*” (Hothorn, Bretz, Westfall, & Heiberger, 2008). Models were only developed for species with more than 30 captures; hence, a total of 15 species were analyzed.

#### 2.5.2 Seasonal differences in the relative importance of local vegetation structure vs landscape‐scale metrics as predictors of bat abundance

To examine the relative effects of local vegetation structure and landscape‐scale metrics in shaping bat abundance patterns, we again used Poisson's GLMMs. Separate sets of models were performed for each focal scale and for each season. In all models, abundance of a given species (number of individuals captured per species) was used as dependent variable and local vegetation structure (PCA1) and landscape metrics as predictors. As above, site nested within location was included as a random effect, and log(effort) was included as an offset. Using variance inflation factors or pairwise Pearson's correlations to a priori exclude highly multicollinear predictor variables from the analysis was not feasible in our case as this would have precluded meaningful comparisons between spatial scales. Hence, we built GLMMs using all nine predictor variables. As argued by Smith, Koper, Francis, and Fahrig (2009), the inclusion of correlated predictors—in our case for instance the different compositional metrics—in the analysis is preferable over removing them as each predictor represents a specific ecological mechanism that potentially influences bat abundance and discarding one of them could lead to biased estimates of the relative importance for the remaining predictors. To ensure robustness of the results, models were only performed for species of which more than 30 individuals were captured per season. This resulted in models for eight species. We ran all predictor subsets models with the “AICcmodavg” package (Mazerolle, 2016) and selected the best‐fit models using Akaike's information criterion corrected for small sample sizes (AIC_c_). Models were retained as best‐fit models when ∆AIC_c_ ≤ 2, that is, when the difference from the best model was ≤2 (Burnham & Anderson, 2002). Model averaging was used to obtain the parameter estimates of the predictors when more than one model had ∆AIC_c_ ≤ 2. Finally, to determine the relative importance of each explanatory variable, we performed a hierarchical partitioning analysis using the “hier.part” package in R (Mac Nally & Walsh, 2004), modified to incorporate a model offset—log(effort) (Jeppsson, Lindhe, Gärdenfors, & Forslund, 2010). Following Benchimol and Peres (2015) and Rocha, Virtanen, and Cabeza (2015), hierarchical partitioning analysis was conducted only considering the fixed effects. To address the issue of potential spatial autocorrelation, the residuals of our best‐fit GLMMs were inspected using the Moran's *I* test. Additionally, an estimate of overdispersion based on the approximately appropriate χ^2^ distribution of the ratio between the sum of squared Pearson's residuals and the residual degrees of freedom was also calculated to assess the quality of the model fit (Bolker et al., 2009). For the majority of the models, no spatial autocorrelation was found (Table S2) and none of the models showed signs of overdispersion (Table S3).

To assess how consistently predictor variables were selected between seasons, we calculated a model consistency index, which measured the agreement of the variables and directions of effects among seasons (Gutzwiller & Barrow, 2001). High interseasonal variation in species‐landscape relations represents low model consistency and vice versa. Following Bonthoux, Barnagaud, Goulard, and Balent (2013), model consistency was calculated as the number of common variables with the same direction of effect between the dry season and the wet season, divided by the total number of landscape variables contained in the best‐fit models.

All analyses were conducted in R v3.1.3 software (R Development Core Team, 2013).

## 3 Results

Based on a total sampling effort of 18,650 mnh, 10,726 mnh in the wet season and 7924 mnh in the dry season, we captured 3,823 phyllostomids and 272 *P. parnellii*. Of those, 1,799 phyllostomids representing 39 species and five subfamilies, as well as 114 *P. parnellii* were captured in the dry season, whereas 2,028 phyllostomids from 41 species and five subfamilies, and 158 *P. parnelli* were caught in the wet season. Only six species were not captured in both seasons (Table S4): *Carollia castanea* and *Micronycteris schmidtorum*—only captured during the dry season—and *Glyphonycteris sylvestris, Lampronycteris brachyotis, Phyllostomus hastatus,* and *Vampyressa pusilla*—only captured during the wet season. Fifty‐six captures, 25 in the dry season and 31 in the wet season, corresponded to individuals recaptured at the same site in the same season and were not considered in the analysis.

### 3.1 Influence of season and habitat type on bat abundance patterns

**Figure 2:**
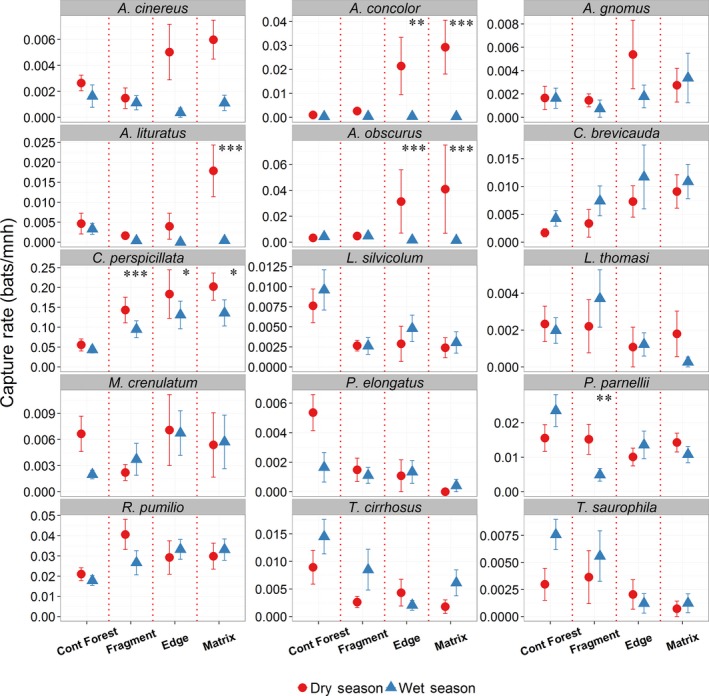
Comparison of mean (±SE) capture rate (bats/mnh) between seasons across different habitat types in the BDFFP landscape. Significant seasonal differences in capture rates based on multiple pairwise comparisons are indicated as ***p < .001, **p < .01, and *p < .05

Species abundances varied widely between seasons and habitat types (Figure 2). Of the 15 species analyzed, 11 showed a significant effect for the season × habitat type interaction (Table S5). Of these 11 species, only five (*Artibeus concolor, A. obscurus, A. lituratus, C. perspicillata,* and *P. parnellii*) showed significant seasonal differences based on multiple pairwise comparisons (Figure 2; Table S6). Seasonal differences in abundances were evident across all habitat types. The abundance of *C. perspicillata* was significantly higher in the dry season for all the three modified habitat types (fragment, edge, and matrix sites). *Artibeus concolor* and *A. obscurus* showed differences in abundance only for edge and matrix sites, with higher capture rates in the dry season for both habitat types. *Artibeus lituratus* and *P. parnellii* had higher capture rates in the dry season for matrix and fragment sites, respectively.

### 3.2 Seasonal responses to local and landscape‐scale predictors

**Figure 3:**
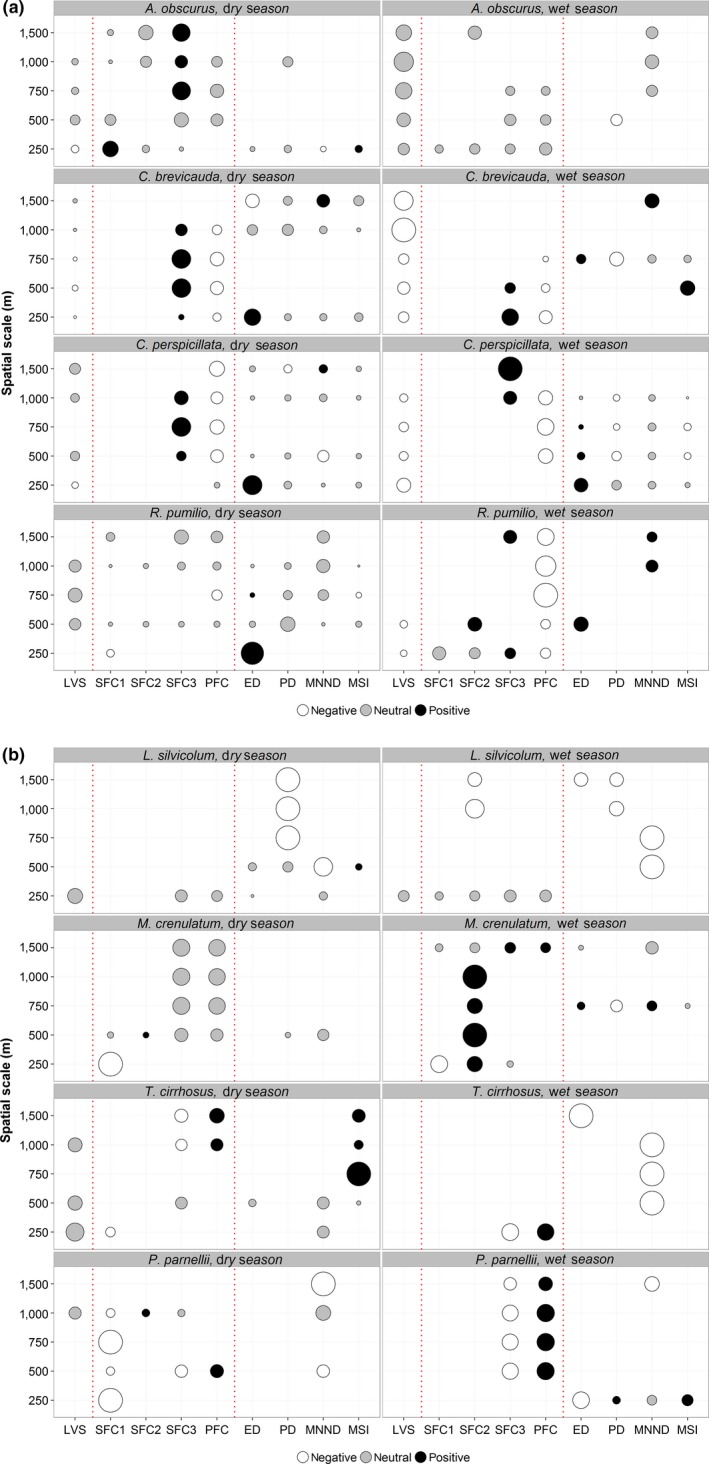
Variation explained by local‐ and landscape‐scale attributes for each combination of season and spatial scale for eight bat species captured in the BDFFP landscape. (a)—frugivores: *Artibeus obscurus*,* Carollia perspicillata*,* C. brevicauda*,* Rhinophylla pumilio*; (b)—animalivores: *Lophostoma silvicolum*,* Mimon crenulatum*,* Trachops cirrhosus*,* Pteronotus parnellii*. Circle size is proportional to the percentage independent contribution of the respective predictor variable to explaining species abundance as determined by hierarchical partitioning. Color represents the direction of the relationship based on the unconditional 95% CIs of the most parsimonious generalized linear mixed models (∆AIC_c_ < 2), where neutral represents a nonsignificant effect and positive/negative represents a significant effect and its respective direction. In each panel, local vegetation structure (LVS), compositional landscape metrics (PFC—primary forest cover; SFC1—initial secondary forest cover; SFC2—intermediate secondary forest cover; SFC3—advanced secondary forest cover) and configurational landscape metrics (ED—edge density; PD—patch density; MNND—mean nearest‐neighbor distance; MSI—mean shape index) are separated by vertical dotted lines. See Tables S8 and S9 for additional modelling results

The relative contributions of local vegetation structure and landscape characteristics to explaining bat abundance responses were both season specific and scale dependent (Figure 3). Compositional metrics were overall more important in the dry season, whereas local scale and configurational metrics played a more important role in the wet season. The way that species responded to these metrics varied between frugivorous and animalivorous species. Frugivores showed a stronger association with compositional metrics in the dry season, with the exception of *R. pumilio*, which showed a strong association with configurational metrics. In the wet season, responses were very variable, with some species responding more to local vegetation structure (*A. obscurus* and *C. brevicauda*) and others responding more to configurational and compositional metrics (*C. perspicillata* and *R. pumilio*). Most animalivorous species showed similar patterns in both seasons, having a strong association with either compositional (*M. crenulatum* and *P. parnellii*) or configurational metrics (*L. silvicolum*) in both seasons. The only exception was *T. cirrhosus*, which responded mostly to configurational metrics in the wet season, whereas in the dry season, it showed relationships with local vegetation structure, compositional and configurational metrics.

**Figure 4:**
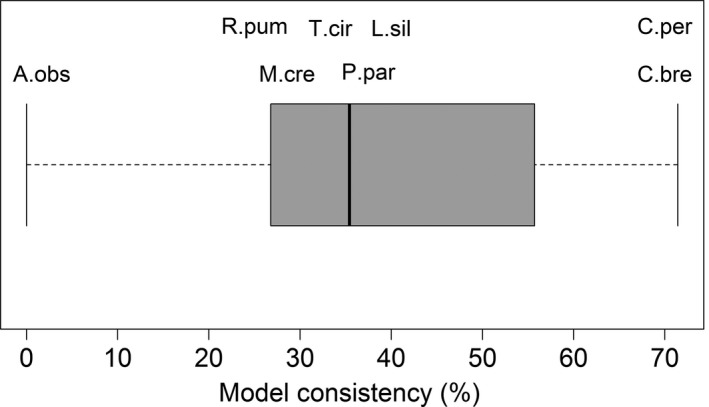
Box‐ and‐whisker‐plot showing the percentage of model consistency between seasons for bat–landscape relationships for eight species of bats (A. obs—*A. obscurus*; C. per—*C. perspicillata*; C. bre—*C. brevicauda*; L. sil—*L. silvicolum*; M. cre—*M. crenulatum*; R. pum—*R. pumilio* T. cir—*T. cirrhosus*; P. par—*P. parnellii*)

A metric‐specific analysis revealed that within compositional and configurational metrics, patterns were very variable, with frugivorous species representing the group with larger variation in model consistency between seasons. Model consistency values averaged 38.4% (*SD* = 23.8) for all eight species, 42% (*SD* = 35.5) for the frugivores, and 34.9% (*SD* = 5) for animalivores. However, values ranged widely from 0% (no common landscape metrics and direction of effects between seasons—*A. obscurus*) to 71% (more than half of the landscape components and direction of effects in common between seasons—*Carollia* spp.; Figure 4; Table S7).

Frugivorous species responded always negatively to PFC and positively to SFC3, whereas animalivores tended to respond positively to PFC and negatively to SFC3 (Table S8). *Mimon crenulatum* was the exception, showing a positive association with both metrics in the wet season and a strong positive association with SFC2 in the same season. In relation to configurational landscape metrics, frugivorous species responded, in general, positively to ED and MNND, while animalivorous species responded negatively to both metrics. *Mimon crenulatum* once again was an exception as it was positively associated with both metrics in the wet season.

General patterns as to which metric was most important at each spatial scale were hard to identify (Figure 3). Different compositional and configurational landscape metrics were selected at all scales for both ensembles without any clearly discernible patterns. On the other hand, local vegetation structure was more consistently selected across all scales for frugivorous species.

## 4 Discussion

### 4.1 Influence of season and habitat type on bat abundance patterns

Capture rates were variable between seasons, with some species showing a clear seasonal pattern. Seasonal differences in abundance occurred mostly in modified habitats (fragments, edge and matrix). In continuous *terra firme* forest fruiting pulses usually occur in the early wet season (Haugaasen & Peres, 2005) and consequently declines in frugivore abundances in primary forest are expected during the dry season (Ortêncio‐Filho, Lacher, & Rodrigues, 2014). The reduction in food availability can lead to a shift of frugivores from primary to secondary forest, where fruit availability can be less seasonal (Barlow, Mestre, Gardner, & Peres, 2007). Bentos, Mesquita, and Williamson (2008) showed that at the BDFFP some *Cecropia* spp. and *Vismia* spp., which are the dominant pioneer trees in the secondary forest matrix, have their flowering and fruiting peaks during the dry season. Due to greater food availability, secondary forest may be a more suitable habitat for some small generalist frugivores (Faria, 2006; de la Peña‐Cuéllar, Stoner, Avila‐Cabadilla, Martínez‐Ramos, & Estrada, 2012), which could lead to a change in their preferred foraging habitat during the dry season. For instance, research showed that monkeys and birds shift their foraging habitat to secondary forest when resources in mature forest become scarce (Bowen, McAlpine, House, & Smith, 2007). Despite this, none of the frugivorous bats showed an increase in capture rates in continuous forest or in the fragments during the wet season, when food availability is higher. An increase in fruit availability in other forest types (e.g., *várzea* forest) in comparison with *terra firme* forests (Haugaasen & Peres, 2005; Ramos Pereira et al., 2010) could explain the absence of this pattern. Bobrowiec, Rosa, Gazarini, and Haugaasen (2014) showed that a drop in the abundance of *Carollia* spp. in *terra firme* and a simultaneous increase in abundance of the same species in *várzea* could indicate seasonal movements between these different forest types. Several studies on birds also have documented a dominant effect of food availability on habitat selection (Naoe, Sakai, Sawa, & Masaki, 2011). Therefore, such inter‐habitat movements to other areas in the landscape could mask the predicted increase in bat abundances in primary forest during the wet season.

### 4.2 Seasonal responses to local and landscape‐scale predictors

Seasonality affected the abundance responses of bats to local and landscape metrics, with both groups of metrics playing an important yet highly variable role between seasons, as suggested by the results of model consistency (Figure 4, Table S7). The relative importance of different landscape predictors and the magnitude of their effect was dependent on the season and species, in agreement with the findings of Cisneros, Fagan and Willig (2015a) and Klingbeil and Willig (2010). Similarly, Vergara and Marquet (2007) showed that the magnitude of the effects of landscape metrics in a bird species were dependent on season. Landscape‐scale characteristics were overall more important than local vegetation structure in modulating bat abundance responses. Contrary to expectations, local vegetation structure only had a greater role than landscape structure in the wet season for two species.

Even though fragment‐matrix contrast at the BDFFP is low and distances between fragments and continuous forest are relatively small, species are influenced by environmental filters that benefit bat species depending on their functional traits (Farneda et al., 2015) and these filters likely differ between seasons. In agreement with studies on understory birds in Atlantic rainforest (Banks‐Leite, Ewers, & Metzger, 2013) and on phyllostomid bats in tropical dry forest (Avila‐Cabadilla, 2012), local and landscape scale characteristics were important at the ensemble and species level. Frugivores and animalivores responded differently to local, compositional, and configurational metrics and no clear patterns regarding responses at different spatial scales emerged. Further analyses are needed to ascertain which scale may be more important for each species/season. However, in a parallel study conducted at the BDFFP, which used the same dataset, yet focused on responses at the assemblage level, Rocha et al. (2017) showed that the direction of effect for total abundance was scale dependent, with for example, total abundance being positively correlated with edge density at the smallest spatial scales and negatively correlated at larger scales.

#### 4.2.1 Frugivore ensemble

In the dry season, frugivores responded more to compositional metrics, whereas during the wet season, local and configurational metrics were more important. *Rhinophylla pumilio* was an exception as it showed the opposite pattern. During the dry season, fruit availability in secondary forest may be higher than in primary forest (Bentos et al., 2008; Haugaasen & Peres, 2005; Ortêncio‐Filho et al., 2014), influencing the responses of frugivores that rely on these resources. All frugivores were positively associated with advanced secondary forest cover (SFC3, age ≥16 years) and negatively associated with primary forest cover (PFC), supporting the assumption that some generalist frugivores prefer regrowth forests as foraging habitat in fragmented landscapes (Klingbeil & Willig, 2009, 2010).

For *R. pumilio,* overall, all configurational metrics were important during the dry season, with abundance being positively associated with edge density at small scales. This suggests that although it can exploit resources in secondary forest, the spatial organization of primary forest patches, and distance between them play an important role. These could be related to the small home range (2.5–16.9 ha) of this species (Henry & Kalko, 2007) and to the fact that small‐scale edges can provide more foraging opportunities and improve connectivity between roosting and foraging areas (Meyer & Kalko, 2008b; Rocha et al., 2017). In the wet season, *R. pumilio* responded more to compositional metrics. Female bats lactate at the onset and during the rainy season (Durant, Hall, Cisneros, Hyland, & Willig, 2013; Henry & Kalko, 2007), increasing their food intake (Henry & Kalko, 2007). Hence, during this period, bats will be more dependent on food availability and distribution, as reflected in a stronger response to compositional metrics.

*Carollia perspicillata* was the only species that responded more to landscape composition (negatively to PFC and positively to SFC3) than to local vegetation structure and configurational metrics in both wet and dry season. In a study conducted in a fragmented landscape characterized by continuous forest surrounded by a matrix of agriculture, development, and logging areas, in unflooded (*terra firme*) Amazonian rainforest, Klingbeil and Willig (2010) found a consistent negative response to primary forest (indicating a preference for secondary forest), regardless of season, for this species. In our study, it represented more than 50% of all bat captures (Table S4), demonstrating its success in exploiting the resources of secondary forest throughout the year. Fruit preferences can influence the foraging behavior of species and therefore can affect how they respond to landscape structure. *Carollia perspicillata* incorporates great proportions of *Vismia* and *Cecropia* (the dominant tree genera in the BDFFP matrix) in its diet (Fleming, 2004), explaining why its abundance was positively influenced by the amount of secondary forest present in the landscape.

Due to higher fruit availability during the wet season, bats do not need to travel long distances for foraging and consequently may respond predominantly to local‐scale characteristics. Cisneros, Fagan and Willig (2015a) found that landscape metrics influenced the metacommunity structure of the frugivore ensemble only in the dry season and suggested that other metrics (e.g., environmental characteristics at the local scale) could be more important in the wet season. Our findings for both *A. obscurus* and *C. brevicauda* are in line with this and demonstrate that local vegetation structure may play a more important role in the wet season for these two species. In the wet season, pregnant and lactating females can reduce their flight durations between foraging and roosting sites in order to compensate for the metabolic cost of producing milk or the increased weight of carrying a fetus (Charles‐Dominique, 1991; Klingbeil & Willig, 2010). Moreover, males of some bat species (e.g., *A. jamaicensis*,* C. perspicillata*) invest time and energy defending roosts and harems during the breeding season (Kunz & Hood, 2000), which could result in smaller home ranges due to the higher energetic demands (Klingbeil & Willig, 2010).

#### 4.2.2 Animalivore ensemble

In contrast to frugivores, animalivores showed a more similar pattern between seasons. Three species responded to the same group of metrics in both seasons, *L. silvicolum* to configuration and *P. parnellii* and *M. crenulatum* to composition, suggesting that for animalivores, seasonality and consequently the variability in resource availability may not play such an important role as it does for frugivores. This contrasts with the findings of Klingbeil and Willig (2010), who found that abundance responses of animalivores to landscape structure differed between seasons, responding to landscape configuration in the dry season and to landscape composition in the wet season. Their study was conducted in a more heterogeneous landscape, whereas the primary forest fragments at the BDFFP are surrounded by a more homogeneous matrix of secondary forest. The observed contrasting patterns in the seasonal response of animalivorous bats to configurational metrics between our study and Klingbeil and Willig (2010) might therefore relate to unequal spatiotemporal distribution of resources across the two study areas.

In the neotropics, abundance of frugivores generally increases in fragmented or disturbed areas, whereas gleaning animalivores tend to decline (Meyer et al., 2016). Although late successional secondary can have structural similarities to primary forest (Ferreira & Prance, 1999), it can take decades or even centuries to resemble old‐growth forests (Guariguata & Ostertag, 2001). In our study landscape, most of the secondary forest in the matrix is <30 years old (Carreiras et al., 2014) and consequently structurally less complex than adjacent continuous forest, constituting less suitable habitat for most gleaning animalivores due to insufficient roosting and prey resources (Meyer & Kalko, 2008b). Therefore, most species will not be able to exploit the seasonal resource peaks that can occur in secondary forest and will be more dependent on primary forest. Accordingly, with the exception of *M. crenulatum*, all animalivorous species showed a negative association with secondary forest cover, edge density, and mean nearest‐neighbor distance in both seasons.

*Trachops cirrhosus* was the only animalivore that showed seasonal variation in abundance, responding more to configurational metrics in the wet season. Responses to configurational metrics may be expected to usually occur during the season when food availability is lower, because bats need to visit habitat of lower quality (e.g., edges) and thus will be more dependent on the spatial arrangement of forest patches (Klingbeil & Willig, 2010). *Trachops cirrhosus* is a gleaning animalivore that feeds mainly on small vertebrates, especially frogs, and insects (Rodrigues, Reis, & Braz, 2004). In the Central Amazon, the wet season is the period of highest frog abundance and juvenile recruitment (Menin, Waldez, & Lima, 2008). Despite this, *T. cirrhosus* showed a greater dependence on configurational metrics in the wet season, suggesting that although frogs are consumed by the species at the BDFFP (Rocha, Gordo, & Lópex‐Baucells, 2016; Rocha, Silva, Marajó Dos Reis, & Rosa, 2012), this prey group may not be as important in this area. Alternatively, fragmentation could be affecting the phenology of its prey, leading to changes in its dietary habits. Changes in dietary habitats in fragmented landscapes due to reduced availability of high‐value food resources have been documented for other taxa such monkeys (Chaves, Stoner, Arroyo‐Rodríguez, & Estrada, 2011). However, further studies are needed in order to understand whether fragmentation is really affecting the dietary habits of *T. cirrhosus*.

## 5 Conclusion

Our results show that seasonality affected the responses of bat species to local vegetation structure and landscape characteristics. This has important implications for the interpretation of models developed to conceptualize species responses to human‐modified landscapes (e.g., Driscoll, Banks, Barton, Lindenmayer, & Smith, 2013; and Villard & Metzger, 2014). Namely it suggests that (1) conceptual models should explicitly account for seasonal differences in species responses to landscape composition and configuration and (2) models should include both local‐scale and landscape‐scale attributes. The importance of the latter point is emphasized by recent evidence showing that the consideration of both patch‐scale and landscape‐scale disturbance variables can lead to dramatically different perceptions regarding the impact of forest modification (Barlow et al., 2016).

Overall, local‐scale metrics were not as important as landscape‐scale metrics; however, for some species, local vegetation structure modulated the ecological responses to fragmentation during the wet season. Forest fragmentation alters the magnitude of seasonal changes in resource availability, causing shifts in foraging strategy, and consequently the scale at which species respond to landscape characteristics, that are probably not observed in unfragmented landscapes (Klingbeil & Willig, 2010). Hence, it is necessary to understand how individual species exploit their habitat and how their dietary habits are jointly affected by fragmentation and seasonality, especially as synergistic effects between fragmentation and seasonality may trigger cascading effects in bat–plant interactions, either directly via seed dispersal and pollination or indirectly via the control of herbivorous arthropods.

Our results indicate that management and conservation efforts should first and foremost focus on preserving landscape‐scale habitat integrity due to the greater contribution of landscape structure in explaining bat abundance responses to fragmentation. This is of critical relevance in landscapes where there is a sharper contrast between forest and matrix (e.g., more heterogeneous landscapes or landscapes with higher anthropogenic pressures) due to the expected increasing contribution of landscape structure characteristics. However, patch‐scale vegetation characteristics can also be important, and therefore, preserving structural habitat integrity at the patch scale in fragmented landscapes should be considered (e.g., preservation of fragments of sufficient size (>100 ha) to minimize detrimental edge effects which degrade smaller forest fragments). This is especially important when food resources are scarce or when bats have reduced home range (e.g., pregnant or lactating females). The idiosyncratic responses of species to seasonal variation in resource abundance and consequently to local and landscape scale attributes means that bat conservation in fragmented landscapes requires multiscale management efforts that encompass both local and landscape scales.

## Conflict of Interest

None declared.

## Author Contributions

C.F.J.M and R.R. conceived the ideas; R.R., A.L.‐B., F.Z.F, J.M.P., and C.F.J.M collected the data; J.M.B.C. conducted the image processing and land cover classification of the study area; D.F.F. analyzed the data and led the writing. All authors discussed the results and commented on the manuscript.
