# Supplementary material for: Season‐modulated responses of Neotropical bats to forest fragmentation
Source: Ecol Evol. 2017 May 13;7(11):4059–71. doi: 10.1002/ece3.3005 (PMC5468172; doi:10.1002/ece3.3005)
Supplement: Supplementary file 1 [file ECE3-7-4059-s001.docx]

**Electronic Supplemental Material**

**Season-modulated responses of Neotropical bats to forest fragmentation**

Diogo F. Ferreira^*^. Ricardo Rocha. Adrià López-Baucells. Fábio Z. Farneda. João M. B. Carreiras. Jorge M. Palmeirim and Christoph F.J. Meyer

^*^**Corresponding author:** Diogo F. Ferreira; **E-mail:** [ferreiradfa@gmail.com](mailto:ferreiradfa@gmail.com)

**This supplementary material contains:**

**Fig. S1** Principal components analysis summarizing vegetation structure

**Fig. S2** Map showing the distribution of the different successional stages of secondary forest at the BDFFP

**Table S1** Variable loadings. eigenvalues and proportion of variance explained by the first two axes of a Principal Component Analysis on the local vegetation structure descriptors

**Table S2** Results of Moran's I test for the residuals of the best-fit GLMMs

**Table S3** Results for the estimate of overdispersion of the best-fit GLMMs

**Table S4** Number of captures for each bat species sampled

**Table S5** Results of likelihood ratio tests comparing the abundance of each species between seasons and habitat types

**Table S6** Results of multiple pairwise comparisons of GLMMs testing for differences in abundance of eleven species between seasons and across habitat types

**Table S7** Results of model consistency between dry and wet season for bat-landscape relationships

**Table S8** Summary results of model averaging of the best-fit GLMMs investigating relationships between bat species abundance and local and landscape-scale attributes

**Table S9** Best-fit models investigating relationships between bat species abundance and local and landscape-scale attributes

**
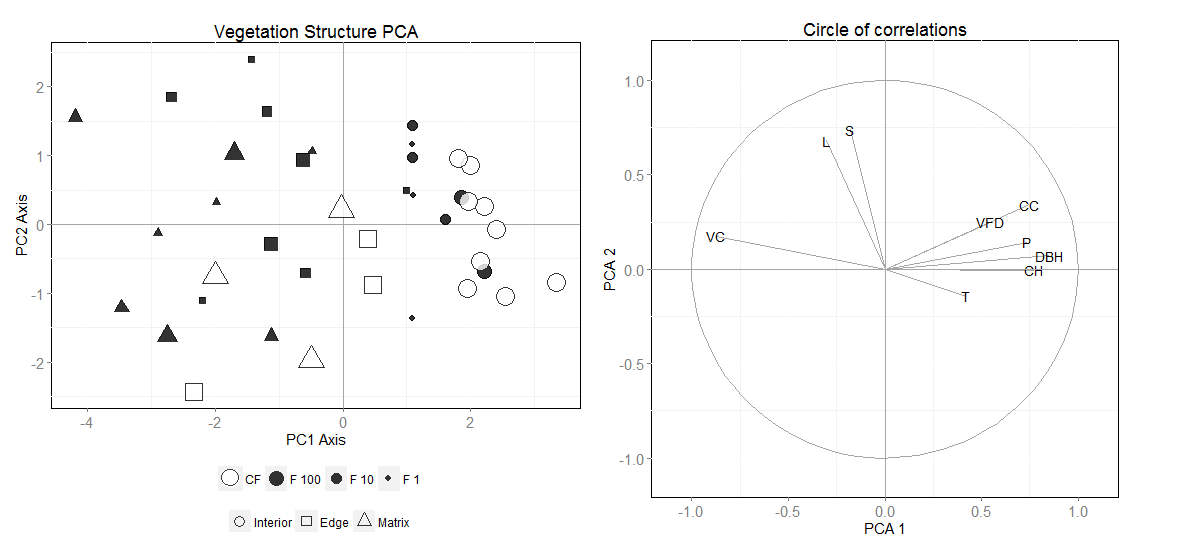
Fig. S1** Principal components analysis examining the covariation between local vegetation structure variables. Variable abbreviations: CF = Continous Forest. F 100 = 100 ha Fragment. F 10 = 10 ha Fragment. F 1 = 1 ha Fragment. CC = canopy cover (%). CH = canopy height (m). DBH = average of the DBH measures of trees ≥10 cm. L = number of lianas. P = number of palms. S = number of woody stems (DBH <10 cm). T = number of trees (DBH >10). VC = number of *Vismia* and *Cecropia* trees. VFD = vertical foliage density (%)





**Fig. S2** Map showing the distribution of the different successional stages of secondary forest around each fragment and continuous forest reserve in the Biological Dynamics of Forest Fragments Project (BDFFP) study area, central Amazon. White represents primary forest cover; different shades of green (from light to dark) represent the different secondary forest cover (SFC) age classes (initial [≤ 5 years] – SFC1, intermediate [6-15 years] – SFC2, advanced [≥ 16 years] – SFC3). The red squares represent the sampling sites

**Table S1** Variable loadings, eigenvalues and proportion of variance explained by the first two axes of a principal components analysis on the local vegetation structure descriptors. Variable abbreviations: CC = canopy cover (%), CH = canopy height (m), DBH = average of the DBH measures of trees ≥10 cm, L = number of lianas, P = number of palms, S = number of woody stems (DBH <10 cm), T = number of trees (DBH >10), VC = number of *Vismia* and *Cecropia* trees, VFD = vertical foliage density (%)

| **Vegetation structure variables** | **PCA 1** | **PCA 2** |
| --- | --- | --- |
| CC | 0.386 | 0.303 |
| S | -0.092 | 0.657 |
| T | 0.216 | -0.128 |
| P | 0.379 | 0.126 |
| L | -0.157 | 0.605 |
| VC | -0.448 | 0.156 |
| DBH | 0.436 | 0.064 |
| CH | 0.399 | -0.005 |
| VFD | 0.280 | 0.222 |
| **Eigenvalue** | 3.8 | 1.3 |
| **% explained** | 42.02 | 14.2 |
| **Cumulative proportion explained** | 42.02 | 56.04 |

**Table S2** Moran’s *I* values of the model residuals of the best-fit GLMMs (Akaike differences <2 from the best model) investigating the relationship between local and landscape-scale attributes on abundance of eight species between the wet and dry seasons and for five focal scales across the BDFFP, Central Amazon. Significant (P < 0.05) results are highlighted in bold. Predictor abbreviations: PFC - primary forest cover; SFC1 - initial secondary forest cover (≤ 5 years); SFC2 - intermediate secondary forest cover (6-15 years); SFC3 - advanced secondary forest cover (≥ 16 years); ED - edge density; PD - patch density; MNND - mean nearest neighbour distance; MSI - mean shape index.

| *Artibeus obscurus* | | | | | | |
| --- | --- | --- | --- | --- | --- | --- |
| **Season** | **Spatial Scale (m)** | **Model structure** | ***I*** | **E(*I*)** | **sd(*I*)** | **p-value** |
| **Dry Season** | **250** | SFC1 | 0.0621 | -0.0263 | 0.0980 | 0.3671 |
|  |  | LVS+SFC1+SFC2+SFC3+ED+PD+MNND+MSI | -0.0413 | -0.0263 | 0.0969 | 0.8774 |
|  |  | ED | -0.0379 | -0.0263 | 0.0971 | 0.9053 |
|  |  | PD | -0.0359 | -0.0263 | 0.0975 | 0.9218 |
|  | **500** | SFC3 | 0.0804 | -0.0263 | 0.0982 | 0.2771 |
|  |  | LVS | 0.0374 | -0.0263 | 0.0978 | 0.5149 |
|  |  | PFC | 0.0635 | -0.0263 | 0.0980 | 0.3594 |
|  |  | SFC1 | 0.0429 | -0.0263 | 0.0978 | 0.4788 |
|  | **750** | SFC3 | 0.0815 | -0.0263 | 0.0981 | 0.2713 |
|  |  | PFC | 0.0748 | -0.0263 | 0.0979 | 0.3015 |
|  |  | LVS | 0.0374 | -0.0263 | 0.0978 | 0.5149 |
|  | **1000** | SFC3 | 0.0889 | -0.0263 | 0.0980 | 0.2397 |
|  |  | SFC1+SFC2+SFC3 | 0.0491 | -0.0263 | 0.0975 | 0.4393 |
|  |  | LVS | 0.0374 | -0.0263 | 0.0978 | 0.5149 |
|  |  | PD | -0.0639 | -0.0263 | 0.0970 | 0.6987 |
|  |  | PFC | 0.0754 | -0.0263 | 0.0978 | 0.2984 |
|  | **1500** | SFC1+SFC2+SFC3 | 0.0494 | -0.0263 | 0.0968 | 0.4342 |
| **Wet Season** | **250** | SFC2 | -0.1308 | -0.0263 | 0.0981 | 0.2870 |
|  |  | LVS | -0.1717 | -0.0263 | 0.0976 | 0.1363 |
|  |  | PFC | -0.1621 | -0.0263 | 0.0979 | 0.1654 |
|  |  | SFC1 | -0.1453 | -0.0263 | 0.0981 | 0.2253 |
|  |  | SFC3 | -0.1608 | -0.0263 | 0.0979 | 0.1697 |
|  | **500** | PD | -0.1036 | -0.0263 | 0.0980 | 0.4305 |
|  |  | LVS | -0.1717 | -0.0263 | 0.0976 | 0.1363 |
|  |  | SFC3 | -0.1650 | -0.0263 | 0.0980 | 0.1568 |
|  |  | PFC | -0.1562 | -0.0263 | 0.0981 | 0.1854 |
|  | **750** | LVS | -0.1717 | -0.0263 | 0.0976 | 0.1363 |
|  |  | MNND | -0.1421 | -0.0263 | 0.0980 | 0.2374 |
|  |  | PFC | -0.1474 | -0.0263 | 0.0982 | 0.2175 |
|  |  | SFC3 | -0.1512 | -0.0263 | 0.0982 | 0.2034 |
|  | **1000** | LVS | -0.1717 | -0.0263 | 0.0976 | 0.1363 |
|  |  | MNND | -0.1548 | -0.0263 | 0.0980 | 0.1902 |
|  | **1500** | SFC2 | -0.1100 | -0.0263 | 0.0979 | 0.3929 |
|  |  | LVS | -0.1717 | -0.0263 | 0.0976 | 0.1363 |
|  |  | MNND | -0.1503 | -0.0263 | 0.0981 | 0.2061 |
| *Carollia brevicauda* | | | | | | |
| **Season** | **Spatial Scale (m)** | **Model structure** | ***I*** | **E(*I*)** | **sd(*I*)** | **p-value** |
| **Dry Season** | **250** | PFC | -0.0269 | -0.0263 | 0.0983 | 0.9950 |
|  |  | LVS | -0.0259 | -0.0263 | 0.0982 | 0.9966 |
|  |  | SFC3 | -0.0115 | -0.0263 | 0.0984 | 0.8800 |
|  |  | LVS+ED+PD+MNND+MSI | -0.0879 | -0.0263 | 0.0976 | 0.5285 |
|  |  | ED | 0.0002 | -0.0263 | 0.0973 | 0.7853 |
|  | **500** | LVS | -0.0259 | -0.0263 | 0.0982 | 0.9966 |
|  |  | PFC | 0.0069 | -0.0263 | 0.0983 | 0.7356 |
|  |  | SFC3 | 0.0075 | -0.0263 | 0.0985 | 0.7312 |
|  | **750** | LVS | -0.0259 | -0.0263 | 0.0982 | 0.9966 |
|  |  | SFC3 | 0.0269 | -0.0263 | 0.0983 | 0.5884 |
|  |  | PFC | 0.0261 | -0.0263 | 0.0982 | 0.5935 |
|  | **1000** | LVS | -0.0259 | -0.0263 | 0.0982 | 0.9966 |
|  |  | LVS+PFC+ED+PD+MNND+MSI | -0.0023 | -0.0263 | 0.0978 | 0.8061 |
|  |  | SFC3 | 0.0268 | -0.0263 | 0.0982 | 0.5886 |
|  | **1500** | ED+PD+MNND+MSI | 0.0650 | -0.0263 | 0.0974 | 0.3481 |
|  |  | LVS+ED+PD+MNND+MSI | 0.0495 | -0.0263 | 0.0972 | 0.4353 |
| **Wet Season** | **250** | SFC3 | -0.0778 | -0.0263 | 0.0973 | 0.5968 |
|  |  | LVS | -0.0777 | -0.0263 | 0.0976 | 0.5988 |
|  |  | PFC | -0.0694 | -0.0263 | 0.0974 | 0.6583 |
|  | **500** | LVS | -0.0777 | -0.0263 | 0.0976 | 0.5988 |
|  |  | SFC3 | -0.0324 | -0.0263 | 0.0972 | 0.9503 |
|  |  | PFC | -0.0391 | -0.0263 | 0.0973 | 0.8956 |
|  |  | MSI | -0.0646 | -0.0263 | 0.0978 | 0.6957 |
|  | **750** | LVS+PFC+ED+PD+MNND+MSI | -0.0973 | -0.0263 | 0.0969 | 0.4640 |
|  |  | LVS | -0.0777 | -0.0263 | 0.0976 | 0.5988 |
|  |  | LVS+ED+PD+MNND+MSI | -0.1768 | -0.0263 | 0.0970 | 0.1209 |
|  | **1000** | LVS | -0.0777 | -0.0263 | 0.0976 | 0.5988 |
|  | **1500** | MNND | -0.0298 | -0.0263 | 0.0972 | 0.9714 |
|  |  | LVS | -0.0777 | -0.0263 | 0.0976 | 0.5988 |
| *Carollia perspicillata* | | | | | | |
| **Season** | **Spatial Scale (m)** | **Model structure** | ***I*** | **E(*I*)** | **sd(*I*)** | **p-value** |
| **Dry Season** | **250** | LVS+ED+PD+MNND+MSI | -0.1882 | -0.0263 | 0.0934 | 0.0831 |
|  |  | LVS+PFC+ED+PD+MNND+MSI | -0.1716 | -0.0263 | 0.0934 | 0.1199 |
|  |  | PFC | -0.1396 | -0.0263 | 0.0922 | 0.2192 |
|  | **500** | PFC | -0.1179 | -0.0263 | 0.0930 | 0.3247 |
|  |  | LVS+PFC+ED+PD+MNND+MSI | -0.1000 | -0.0263 | 0.0931 | 0.4285 |
|  |  | SFC3 | -0.1090 | -0.0263 | 0.0918 | 0.3679 |
|  | **750** | SFC3 | -0.0982 | -0.0263 | 0.0928 | 0.4388 |
|  |  | PFC | -0.0896 | -0.0263 | 0.0935 | 0.4987 |
|  | **1000** | SFC3 | -0.0805 | -0.0263 | 0.0929 | 0.5599 |
|  |  | LVS+PFC+ED+PD+MNND+MSI | -0.0428 | -0.0263 | 0.0942 | 0.8607 |
|  | **1500** | LVS+PFC+ED+PD+MNND+MSI | -0.1579 | -0.0263 | 0.0947 | 0.1645 |
| **Wet Season** | **250** | LVS+ED+PD+MNND+MSI | -0.1036 | -0.0263 | 0.0878 | 0.3788 |
|  | **500** | LVS+PFC+ED+PD+MNND+MSI | -0.0627 | -0.0263 | 0.0904 | 0.6873 |
|  | **750** | LVS+PFC+ED+PD+MNND+MSI | 0.0094 | -0.0263 | 0.0857 | 0.6770 |
|  | **1000** | PFC | -0.0348 | -0.0263 | 0.0817 | 0.9170 |
|  |  | SFC3 | -0.0265 | -0.0263 | 0.0818 | 0.9980 |
|  |  | LVS+PFC+ED+PD+MNND+MSI | 0.0546 | -0.0263 | 0.0839 | 0.3349 |
|  | **1500** | SFC3 | 0.0041 | -0.0263 | 0.0817 | 0.7098 |
| *Rhinophylla pumilio* | | | | | | |
| **Season** | **Spatial Scale (m)** | **Model structure** | ***I*** | **E(*I*)** | **sd(*I*)** | **p-value** |
| **Dry Season** | **250** | SFC1 | -0.1459 | -0.0263 | 0.0976 | 0.2205 |
|  |  | ED | -0.1476 | -0.0263 | 0.0976 | 0.2138 |
|  | **500** | PD | -0.1060 | -0.0263 | 0.0977 | 0.4150 |
|  |  | SFC2 | -0.1394 | -0.0263 | 0.0975 | 0.2459 |
|  |  | ED | -0.1704 | -0.0263 | 0.0976 | 0.1398 |
|  |  | PFC | -0.1398 | -0.0263 | 0.0975 | 0.2449 |
|  |  | SFC3 | -0.1397 | -0.0263 | 0.0976 | 0.2450 |
|  |  | LVS | -0.1429 | -0.0263 | 0.0977 | 0.2325 |
|  |  | MSI | -0.1365 | -0.0263 | 0.0976 | 0.2591 |
|  |  | SFC1 | -0.1398 | -0.0263 | 0.0976 | 0.2450 |
|  |  | MNND | -0.1391 | -0.0263 | 0.0976 | 0.2479 |
|  | **750** | LVS+PFC+ED+PD+MNND+MSI | -0.0373 | -0.0263 | 0.0974 | 0.9106 |
|  |  | PD | -0.1214 | -0.0263 | 0.0976 | 0.3302 |
|  | **1000** | SFC3 | -0.1358 | -0.0263 | 0.0975 | 0.2614 |
|  |  | PFC | -0.1401 | -0.0263 | 0.0975 | 0.2433 |
|  |  | MSI | -0.1419 | -0.0263 | 0.0975 | 0.2360 |
|  |  | PD | -0.1276 | -0.0263 | 0.0976 | 0.2993 |
|  |  | SFC1 | -0.1335 | -0.0263 | 0.0975 | 0.2716 |
|  |  | ED | -0.1566 | -0.0263 | 0.0975 | 0.1815 |
|  |  | MNND | -0.1376 | -0.0263 | 0.0976 | 0.2540 |
|  |  | SFC2 | -0.1483 | -0.0263 | 0.0976 | 0.2113 |
|  |  | LVS | -0.1429 | -0.0263 | 0.0977 | 0.2325 |
|  | **1500** | SFC3 | -0.1377 | -0.0263 | 0.0975 | 0.2532 |
|  |  | PFC | -0.1417 | -0.0263 | 0.0975 | 0.2366 |
|  |  | SFC1 | -0.1361 | -0.0263 | 0.0974 | 0.2597 |
|  |  | MNND | -0.1268 | -0.0263 | 0.0975 | 0.3028 |
| **Wet Season** | **250** | SFC3 | -0.0788 | -0.0263 | 0.0949 | 0.5801 |
|  |  | PFC | -0.0784 | -0.0263 | 0.0950 | 0.5839 |
|  |  | LVS | -0.1494 | -0.0263 | 0.0967 | 0.2031 |
|  |  | SFC1+SFC2+SFC3 | -0.0942 | -0.0263 | 0.0952 | 0.4757 |
|  | **500** | SFC2 | -0.0794 | -0.0263 | 0.0956 | 0.5784 |
|  |  | PFC | -0.0555 | -0.0263 | 0.0948 | 0.7586 |
|  |  | ED | -0.0797 | -0.0263 | 0.0957 | 0.5768 |
|  |  | LVS | -0.1494 | -0.0263 | 0.0967 | 0.2031 |
|  | **750** | PFC | -0.0350 | -0.0263 | 0.0948 | 0.9274 |
|  | **1000** | PFC | -0.1434 | -0.0263 | 0.0983 | 0.2336 |
|  |  | MNND | -0.1548 | -0.0263 | 0.0980 | 0.1902 |
|  | **1500** | MNND | -0.0843 | -0.0263 | 0.0952 | 0.5422 |
|  |  | PFC | -0.0210 | -0.0263 | 0.0951 | 0.9553 |
|  |  | SFC3 | -0.0372 | -0.0263 | 0.0953 | 0.9088 |
| *Lophostoma silvicolum* | | | | | | |
| **Season** | **Spatial Scale (m)** | **Model structure** | ***I*** | **E(*I*)** | **sd(*I*)** | **p-value** |
| **Dry Season** | **250** | LVS | -0.1155 | -0.0263 | 0.0963 | 0.3543 |
|  |  | SFC3 | -0.1101 | -0.0263 | 0.0964 | 0.3847 |
|  |  | MNND | -0.1349 | -0.0263 | 0.0963 | 0.2598 |
|  |  | PFC | -0.1060 | -0.0263 | 0.0963 | 0.4080 |
|  |  | ED | -0.1084 | -0.0263 | 0.0969 | 0.3966 |
|  | **500** | ED+PD+MNND+MSI | -0.1286 | -0.0263 | 0.0952 | 0.2826 |
|  |  | PD | -0.0919 | -0.0263 | 0.0963 | 0.4958 |
|  | **750** | PD | -0.0922 | -0.0263 | 0.0953 | 0.4894 |
|  | **1000** | PD | -0.1429 | -0.0263 | 0.0952 | 0.2209 |
|  | **1500** | PD | -0.1394 | -0.0263 | 0.0963 | 0.2406 |
| **Wet Season** | **250** | SFC1+SFC2+SFC3 | 0.3260 | -0.0263 | 0.0979 | **0.0003** |
|  |  | SFC2 | 0.3596 | -0.0263 | 0.0983 | **0.0001** |
|  |  | SFC3 | 0.4031 | -0.0263 | 0.0979 | **0.0000** |
|  |  | PFC | 0.4041 | -0.0263 | 0.0980 | **0.0000** |
|  |  | LVS | 0.3954 | -0.0263 | 0.0976 | **0.0000** |
|  |  | PFC+SFC1+SFC2+SFC3 | 0.3190 | -0.0263 | 0.0975 | **0.0004** |
|  | **500** | MNND | 0.3503 | -0.0263 | 0.0975 | **0.0001** |
|  | **750** | MNND | 0.2992 | -0.0263 | 0.0979 | **0.0009** |
|  | **1000** | PD | 0.2680 | -0.0263 | 0.0979 | **0.0026** |
|  |  | SFC2 | 0.2307 | -0.0263 | 0.0983 | **0.0090** |
|  | **1500** | PD | 0.2828 | -0.0263 | 0.0981 | **0.0016** |
|  |  | ED | 0.3492 | -0.0263 | 0.0984 | **0.0001** |
|  |  | SFC2 | 0.2621 | -0.0263 | 0.0985 | **0.0034** |
| *Mimon crenulatum* | | | | | | |
| **Season** | **Spatial Scale (m)** | **Model structure** | ***I*** | **E(*I*)** | **sd(*I*)** | **p-value** |
| **Dry Season** | **250** | SFC1 | 0.0748 | -0.0263 | 0.0952 | 0.2885 |
|  | **500** | SFC3 | 0.0288 | -0.0263 | 0.0959 | 0.5656 |
|  |  | Only SFC Predictors | 0.0547 | -0.0263 | 0.0949 | 0.3931 |
|  |  | MNND | 0.0081 | -0.0263 | 0.0959 | 0.7199 |
|  |  | SFC1 | 0.0364 | -0.0263 | 0.0971 | 0.5180 |
|  |  | PFC | 0.0258 | -0.0263 | 0.0963 | 0.5881 |
|  |  | PD | 0.0012 | -0.0263 | 0.0970 | 0.7764 |
|  | **750** | SFC3 | 0.0335 | -0.0263 | 0.0964 | 0.5350 |
|  |  | PFC | 0.0240 | -0.0263 | 0.0966 | 0.6027 |
|  | **1000** | SFC3 | 0.0377 | -0.0263 | 0.0966 | 0.5073 |
|  |  | PFC | 0.0303 | -0.0263 | 0.0968 | 0.5588 |
|  | **1500** | SFC3 | 0.0391 | -0.0263 | 0.0966 | 0.4984 |
|  |  | PFC | 0.0338 | -0.0263 | 0.0968 | 0.5347 |
| **Wet Season** | **250** | SFC1+SFC2+SFC3 | -0.1551 | -0.0263 | 0.0972 | 0.1851 |
|  | **500** | SFC2 | -0.1047 | -0.0263 | 0.0982 | 0.4245 |
|  | **750** | SFC2 | -0.0948 | -0.0263 | 0.0982 | 0.4855 |
|  |  | ED+PD+MNND+MSI | -0.0823 | -0.0263 | 0.0976 | 0.5660 |
|  | **1000** | SFC2 | -0.1102 | -0.0263 | 0.0982 | 0.3928 |
|  | **1500** | SFC2 | -0.1253 | -0.0263 | 0.0984 | 0.3147 |
|  |  | MNND | -0.0814 | -0.0263 | 0.0981 | 0.5746 |
|  |  | PFC+SFC1+SFC2+SFC3 | -0.0673 | -0.0263 | 0.0980 | 0.6758 |
|  |  | ED | -0.1059 | -0.0263 | 0.0983 | 0.4183 |
|  |  | SFC1 | -0.0996 | -0.0263 | 0.0980 | 0.4550 |
| *Trachops cirrhosus* | | | | | | |
| **Season** | **Spatial Scale (m)** | **Model structure** | ***I*** | **E(*I*)** | **sd(*I*)** | **p-value** |
| **Dry Season** | **250** | SFC1 | -0.0482 | -0.0263 | 0.0980 | 0.8230 |
|  |  | LVS | 0.0115 | -0.0263 | 0.0965 | 0.6947 |
|  |  | MNND | -0.0428 | -0.0263 | 0.0980 | 0.8666 |
|  | **500** | ED | 0.0671 | -0.0263 | 0.0973 | 0.3369 |
|  |  | LVS | 0.0115 | -0.0263 | 0.0965 | 0.6947 |
|  |  | MSI | 0.0621 | -0.0263 | 0.0979 | 0.3662 |
|  |  | MNND | 0.0034 | -0.0263 | 0.0973 | 0.7603 |
|  |  | SFC3 | -0.0305 | -0.0263 | 0.0976 | 0.9659 |
|  | **750** | MSI | 0.0381 | -0.0263 | 0.0971 | 0.5070 |
|  | **1000** | SFC3 | -0.0409 | -0.0263 | 0.0973 | 0.8807 |
|  |  | MSI | 0.0282 | -0.0263 | 0.0973 | 0.5749 |
|  |  | PFC | -0.0419 | -0.0263 | 0.0974 | 0.8730 |
|  |  | LVS | 0.0115 | -0.0263 | 0.0965 | 0.6947 |
|  | **1500** | MSI | -0.0023 | -0.0263 | 0.0971 | 0.8048 |
|  |  | PFC | -0.0383 | -0.0263 | 0.0972 | 0.9018 |
|  |  | SFC3 | -0.0367 | -0.0263 | 0.0971 | 0.9147 |
| **Wet Season** | **250** | SFC3 | -0.1496 | -0.0263 | 0.0979 | 0.2080 |
|  |  | PFC | -0.1451 | -0.0263 | 0.0980 | 0.2257 |
|  | **500** | MNND | -0.0881 | -0.0263 | 0.0981 | 0.5285 |
|  | **750** | MNND | -0.0791 | -0.0263 | 0.0982 | 0.5912 |
|  | **1000** | MNND | -0.1053 | -0.0263 | 0.0980 | 0.4202 |
|  | **1500** | ED | -0.0625 | -0.0263 | 0.0980 | 0.7119 |
| *Pteronotus parnellii* | | | | | | |
| **Season** | **Spatial Scale (m)** | **Model structure** | ***I*** | **E(*I*)** | **sd(*I*)** | **p-value** |
| **Dry Season** | **250** | SFC1 | 0.0527 | -0.0263 | 0.0959 | 0.4095 |
|  | **500** | PFC | 0.1260 | -0.0263 | 0.0963 | 0.1138 |
|  |  | SFC3 | 0.1200 | -0.0263 | 0.0960 | 0.1276 |
|  |  | SFC1 | 0.1153 | -0.0263 | 0.0966 | 0.1426 |
|  |  | MNND | 0.1788 | -0.0263 | 0.0969 | **0.0342** |
|  | **750** | SFC1 | 0.1128 | -0.0263 | 0.0965 | 0.1494 |
|  | **1000** | SFC1 | 0.1282 | -0.0263 | 0.0962 | 0.1084 |
|  |  | SFC1+SFC2+SFC3 | 0.1220 | -0.0263 | 0.0969 | 0.1260 |
|  |  | MNND | 0.1294 | -0.0263 | 0.0969 | 0.1082 |
|  |  | LVS | 0.1128 | -0.0263 | 0.0970 | 0.1516 |
|  | **1500** | MNND | 0.1239 | -0.0263 | 0.0968 | 0.1208 |
| **Wet Season** | **250** | ED+PD+MNND+MSI | 0.0496 | -0.0263 | 0.0970 | 0.4342 |
|  | **500** | PFC | 0.0765 | -0.0263 | 0.0967 | 0.2878 |
|  |  | SFC3 | 0.0527 | -0.0263 | 0.0967 | 0.4136 |
|  | **750** | PFC | 0.0754 | -0.0263 | 0.0966 | 0.2923 |
|  |  | SFC3 | 0.0569 | -0.0263 | 0.0965 | 0.3881 |
|  | **1000** | PFC | 0.0753 | -0.0263 | 0.0966 | 0.2929 |
|  |  | SFC3 | 0.0560 | -0.0263 | 0.0964 | 0.3932 |
|  | **1500** | PFC | 0.0664 | -0.0263 | 0.0966 | 0.3368 |
|  |  | SFC3 | 0.0448 | -0.0263 | 0.0965 | 0.4612 |
|  |  | MNND | 0.0676 | -0.0263 | 0.0968 | 0.3321 |

**Table S3** Results for the estimate of overdispersion of the best-fit GLMMs (Akaike differences < 2 from the best model) investigating the relationship between local and landscape-scale attributes on abundance of eight species between the wet and dry seasons and for five focal scales across the BDFFP, Central Amazon. P-value is based on the approximately appropriate χ2 distribution and P < 0.05 indicates models with signs of over-dispersion. Predictor abbreviations: PFC - primary forest cover; SFC1 - initial secondary forest cover (≤ 5 years); SFC2 - intermediate secondary forest cover (6-15 years); SFC3 - advanced secondary forest cover (≥ 16 years); ED - edge density; PD - patch density; MNND - mean nearest neighbour distance; MSI - mean shape index.

| *Artibeus obscurus* | | | | | | | | | | | | | | | | | | | |
| --- | --- | --- | --- | --- | --- | --- | --- | --- | --- | --- | --- | --- | --- | --- | --- | --- | --- | --- | --- |
| **Season** | **Spatial Scale (m)** | | | **Model structure** | | ***Sum of squared***  ***Pearson residuals*** | **Ratio**  **(SSQ residuals/rdf)** | | | | | | | **Residual df** | | | | **p-value** |  |
| **Dry Season** | **250** | | SFC1 | | | 7.579 | | | | 0.217 | | | 35 | | | | 1 | | |
|  |  |  | LVS+SFC1+SFC2+SFC3+ED+PD+MNND+MSI | | | 29.175 | | | | 1.042 | | | 28 | | | | 0.404 | | |
|  |  |  | ED | | | 7.064 | | | | 0.202 | | | 35 | | | | 1 | | |
|  |  |  | PD | | | 8.134 | | | | 0.232 | | | 35 | | | | 1 | | |
|  | **500** | | SFC3 | | | 7.422 | | | | 0.212 | | | 35 | | | | 1 | | |
|  |  |  | LVS | | | 7.421 | | | | 0.212 | | | 35 | | | | 1 | | |
|  |  |  | PFC | | | 7.437 | | | | 0.212 | | | 35 | | | | 1 | | |
|  |  |  | SFC1 | | | 7.501 | | | | 0.214 | | | 35 | | | | 1 | | |
|  | **750** | | SFC3 | | | 8.227 | | | | 0.235 | | | 35 | | | | 1 | | |
|  |  |  | PFC | | | 7.719 | | | | 0.221 | | | 35 | | | | 1 | | |
|  |  |  | LVS | | | 7.421 | | | | 0.212 | | | 35 | | | | 1 | | |
|  | **1000** | | SFC3 | | | 8.251 | | | | 0.236 | | | 35 | | | | 1 | | |
|  |  |  | SFC1+SFC2+SFC3 | | | 12.834 | | | | 0.389 | | | 33 | | | | 1 | | |
|  |  |  | LVS | | | 7.421 | | | | 0.212 | | | 35 | | | | 1 | | |
|  |  |  | PD | | | 7.492 | | | | 0.214 | | | 35 | | | | 1 | | |
|  |  |  | PFC | | | 7.534 | | | | 0.215 | | | 35 | | | | 1 | | |
|  | **1500** | | SFC1+SFC2+SFC3 | | | 14.957 | | | | 0.453 | | | 33 | | | | 0.997 | | |
| **Wet Season** | **250** | | SFC2 | | | 18.161 | | | | 0.519 | | | 35 | | | | 0.992 | | |
|  |  |  | LVS | | | 19.735 | | | | 0.564 | | | 35 | | | | 0.982 | | |
|  |  |  | PFC | | | 20.204 | | | | 0.577 | | | 35 | | | | 0.978 | | |
|  |  |  | SFC1 | | | 18.679 | | | | 0.534 | | | 35 | | | | 0.989 | | |
|  |  |  | SFC3 | | | 19.807 | | | | 0.566 | | | 35 | | | | 0.982 | | |
|  | **500** | | PD | | | 21.504 | | | | 0.614 | | | 35 | | | | 0.964 | | |
|  |  |  | LVS | | | 19.735 | | | | 0.564 | | | 35 | | | | 0.982 | | |
|  |  |  | SFC3 | | | 19.367 | | | | 0.553 | | | 35 | | | | 0.985 | | |
|  |  |  | PFC | | | 18.621 | | | | 0.532 | | | 35 | | | | 0.989 | | |
|  | **750** | | LVS | | | 19.735 | | | | 0.564 | | | 35 | | | | 0.982 | | |
|  |  |  | MNND | | | 18.720 | | | | 0.535 | | | 35 | | | | 0.989 | | |
|  |  |  | PFC | | | 17.038 | | | | 0.487 | | | 35 | | | | 0.995 | | |
|  |  |  | SFC3 | | | 17.269 | | | | 0.493 | | | 35 | | | | 0.995 | | |
|  | **1000** | | LVS | | | 19.735 | | | | 0.564 | | | 35 | | | | 0.982 | | |
|  |  |  | MNND | | | 17.991 | | | | 0.514 | | | 35 | | | | 0.992 | | |
|  | **1500** | | SFC2 | | | 16.751 | | | | 0.479 | | | 35 | | | | 0.996 | | |
|  |  |  | LVS | | | 19.735 | | | | 0.564 | | | 35 | | | | 0.982 | | |
|  |  |  | MNND | | | 17.967 | | | | 0.513 | | | 35 | | | | 0.992 | | |
| *Carollia brevicauda* | | | | | | | | | | | | | | | | | | | |
| **Season** | **Spatial Scale (m)** | | **Model structure** | | ***Sum of squared***  ***Pearson residuals*** | | | | **Ratio**  **(SSQ residuals/rdf)** | | | **Residual df** | | | | **p-value** | | | |
| **Dry Season** | **250** | | PFC | | 23.942 | | | | 0.684 | | | 35 | | | | 0.921 | | | |
|  |  |  | LVS | | 18.782 | | | | 0.537 | | | 35 | | | | 0.989 | | | |
|  |  |  | SFC3 | | 24.354 | | | | 0.696 | | | 35 | | | | 0.911 | | | |
|  |  |  | LVS+ED+PD+MNND+MSI | | 19.489 | | | | 0.629 | | | 31 | | | | 0.946 | | | |
|  |  |  | ED | | 33.222 | | | | 0.949 | | | 35 | | | | 0.554 | | | |
|  | **500** | | LVS | | 18.782 | | | | 0.537 | | | 35 | | | | 0.989 | | | |
|  |  |  | PFC | | 28.585 | | | | 0.817 | | | 35 | | | | 0.770 | | | |
|  |  |  | SFC3 | | 27.637 | | | | 0.790 | | | 35 | | | | 0.808 | | | |
|  | **750** | | LVS | | 18.782 | | | | 0.537 | | | 35 | | | | 0.989 | | | |
|  |  |  | SFC3 | | 30.774 | | | | 0.879 | | | 35 | | | | 0.672 | | | |
|  |  |  | PFC | | 28.520 | | | | 0.815 | | | 35 | | | | 0.773 | | | |
|  | **1000** | | LVS | | 18.782 | | | | 0.537 | | | 35 | | | | 0.989 | | | |
|  |  |  | LVS+PFC+ED+PD+MNND+MSI | | 33.387 | | | | 1.113 | | | 30 | | | | 0.306 | | | |
|  |  |  | SFC3 | | 29.553 | | | | 0.844 | | | 35 | | | | 0.728 | | | |
|  | **1500** | | ED+PD+MNND+MSI | | 35.002 | | | | 1.094 | | | 32 | | | | 0.327 | | | |
|  |  |  | LVS+ED+PD+MNND+MSI | | 27.451 | | | | 0.886 | | | 31 | | | | 0.649 | | | |
| **Wet Season** | **250** | | SFC3 | | 14.661 | | | | 0.419 | | | 35 | | | | 0.999 | | | |
|  |  |  | LVS | | 15.653 | | | | 0.447 | | | 35 | | | | 0.998 | | | |
|  |  |  | PFC | | 14.649 | | | | 0.419 | | | 35 | | | | 0.999 | | | |
|  | **500** | | LVS | | 15.653 | | | | 0.447 | | | 35 | | | | 0.998 | | | |
|  |  |  | SFC3 | | 14.208 | | | | 0.406 | | | 35 | | | | 0.999 | | | |
|  |  |  | PFC | | 14.741 | | | | 0.421 | | | 35 | | | | 0.999 | | | |
|  |  |  | MSI | | 12.402 | | | | 0.354 | | | 35 | | | | 1 | | | |
|  | **750** | | LVS+PFC+ED+PD+MNND+MSI | | 33.022 | | | | 1.101 | | | 30 | | | | 0.322 | | | |
|  |  |  | LVS | | 15.653 | | | | 0.447 | | | 35 | | | | 0.998 | | | |
|  |  |  | LVS+ED+PD+MNND+MSI | | 21.385 | | | | 0.690 | | | 31 | | | | 0.901 | | | |
|  | **1000** | | LVS | | 15.653 | | | | 0.447 | | | 35 | | | | 0.998 | | | |
|  | **1500** | | MNND | | 17.598 | | | | 0.503 | | | 35 | | | | 0.994 | | | |
|  |  |  | LVS | | 15.653 | | | | 0.447 | | | 35 | | | | 0.998 | | | |
| *Carollia perspicillata* | | | | | | | | | | | | | | | | | | | |
| **Season** | **Spatial Scale (m)** | | **Model structure** | | ***Sum of squared Pearson residuals*** | | | | **Ratio**  **(SSQ residuals/rdf)** | | | **Residual df** | | | | **p-value** | | | |
| **Dry Season** | **250** | | LVS+ED+PD+MNND+MSI | | 9.133 | | | | 0.295 | | | 31 | | | | 1 | | | |
|  |  |  | LVS+PFC+ED+PD+MNND+MSI | | 9.836 | | | | 0.328 | | | 30 | | | | 1 | | | |
|  |  |  | PFC | | 6.862 | | | | 0.196 | | | 35 | | | | 1 | | | |
|  | **500** | | PFC | | 7.730 | | | | 0.221 | | | 35 | | | | 1 | | | |
|  |  |  | LVS+PFC+ED+PD+MNND+MSI | | 11.465 | | | | 0.382 | | | 30 | | | | 0.999 | | | |
|  |  |  | SFC3 | | 7.464 | | | | 0.213 | | | 35 | | | | 1 | | | |
|  | **750** | | SFC3 | | 8.569 | | | | 0.245 | | | 35 | | | | 1 | | | |
|  |  |  | PFC | | 7.939 | | | | 0.227 | | | 35 | | | | 1 | | | |
|  | **1000** | | SFC3 | | 8.698 | | | | 0.249 | | | 35 | | | | 1 | | | |
|  |  |  | LVS+PFC+ED+PD+MNND+MSI | | 11.413 | | | | 0.380 | | | 30 | | | | 0.999 | | | |
|  | **1500** | | LVS+PFC+ED+PD+MNND+MSI | | 12.339 | | | | 0.411 | | | 30 | | | | 0.998 | | | |
| **Wet Season** | **250** | | LVS+ED+PD+MNND+MSI | | 7.652 | | | | 0.247 | | | 31 | | | | 1 | | | |
|  | **500** | | LVS+PFC+ED+PD+MNND+MSI | | 10.447 | | | | 0.348 | | | 30 | | | | 1 | | | |
|  | **750** | | LVS+PFC+ED+PD+MNND+MSI | | 9.050 | | | | 0.302 | | | 30 | | | | 1 | | | |
|  | **1000** | | PFC | | 7.078 | | | | 0.202 | | | 35 | | | | 1 | | | |
|  |  |  | SFC3 | | 7.189 | | | | 0.205 | | | 35 | | | | 1 | | | |
|  |  |  | LVS+PFC+ED+PD+MNND+MSI | | 10.578 | | | | 0.353 | | | 30 | | | | 1 | | | |
|  | **1500** | | SFC3 | | 7.121 | | | | 0.203 | | | 35 | | | | 1 | | | |
| *Rhinophylla pumilio* | | | | | | | | | | | | | | | | | | | |
| **Season** | **Spatial Scale (m)** | | **Model structure** | | ***Sum of squared Pearson residuals*** | | | | **Ratio**  **(SSQ residuals/rdf)** | | | **Residual df** | | | | **p-value** | | | |
| **Dry Season** | **250** | | SFC1 | | 28.881 | | | 0.825 | | | | 35 | | | | 0.757 | | | |
|  |  |  | ED | | 29.962 | | | 0.856 | | | | 35 | | | | 0.710 | | | |
|  | **500** | | PD | | 24.018 | | | 0.686 | | | | 35 | | | | 0.919 | | | |
|  |  |  | SFC2 | | 24.362 | | | 0.696 | | | | 35 | | | | 0.911 | | | |
|  |  |  | ED | | 25.469 | | | 0.728 | | | | 35 | | | | 0.881 | | | |
|  |  |  | PFC | | 23.773 | | | 0.679 | | | | 35 | | | | 0.925 | | | |
|  |  |  | SFC3 | | 23.462 | | | 0.670 | | | | 35 | | | | 0.931 | | | |
|  |  |  | LVS | | 25.203 | | | 0.720 | | | | 35 | | | | 0.889 | | | |
|  |  |  | MSI | | 25.306 | | | 0.723 | | | | 35 | | | | 0.886 | | | |
|  |  |  | SFC1 | | 23.791 | | | 0.680 | | | | 35 | | | | 0.924 | | | |
|  |  |  | MNND | | 24.272 | | | 0.693 | | | | 35 | | | | 0.913 | | | |
|  | **750** | | LVS+PFC+ED+PD+MNND+MSI | | 39.722 | | | 1.324 | | | | 30 | | | | 0.110 | | | |
|  |  |  | PD | | 23.268 | | | 0.665 | | | | 35 | | | | 0.935 | | | |
|  | **1000** | | SFC3 | | 25.887 | | | 0.740 | | | | 35 | | | | 0.869 | | | |
|  |  |  | PFC | | 26.443 | | | 0.756 | | | | 35 | | | | 0.851 | | | |
|  |  |  | MSI | | 24.931 | | | 0.712 | | | | 35 | | | | 0.896 | | | |
|  |  |  | PD | | 23.910 | | | 0.683 | | | | 35 | | | | 0.922 | | | |
|  |  |  | SFC1 | | 26.497 | | | 0.757 | | | | 35 | | | | 0.849 | | | |
|  |  |  | ED | | 24.751 | | | 0.707 | | | | 35 | | | | 0.901 | | | |
|  |  |  | MNND | | 25.566 | | | 0.730 | | | | 35 | | | | 0.878 | | | |
|  |  |  | SFC2 | | 24.423 | | | 0.698 | | | | 35 | | | | 0.910 | | | |
|  |  |  | LVS | | 25.203 | | | 0.720 | | | | 35 | | | | 0.889 | | | |
|  | **1500** | | SFC3 | | 26.859 | | | 0.767 | | | | 35 | | | | 0.836 | | | |
|  |  |  | PFC | | 27.602 | | | 0.789 | | | | 35 | | | | 0.809 | | | |
|  |  |  | SFC1 | | 28.308 | | | 0.809 | | | | 35 | | | | 0.781 | | | |
|  |  |  | MNND | | 22.891 | | | 0.654 | | | | 35 | | | | 0.943 | | | |
| **Wet Season** | **250** | | SFC3 | | 22.281 | | | 0.637 | | | | 35 | | | | 0.953 | | | |
|  |  |  | PFC | | 22.199 | | | 0.634 | | | | 35 | | | | 0.954 | | | |
|  |  |  | LVS | | 21.752 | | | 0.621 | | | | 35 | | | | 0.961 | | | |
|  |  |  | SFC1+SFC2+SFC3 | | 25.198 | | | 0.764 | | | | 33 | | | | 0.833 | | | |
|  | **500** | | SFC2 | | 22.667 | | | 0.648 | | | | 35 | | | | 0.947 | | | |
|  |  |  | PFC | | 22.470 | | | 0.642 | | | | 35 | | | | 0.950 | | | |
|  |  |  | ED | | 21.766 | | | 0.622 | | | | 35 | | | | 0.961 | | | |
|  |  |  | LVS | | 21.752 | | | 0.621 | | | | 35 | | | | 0.961 | | | |
|  | **750** | | PFC | | 23.462 | | | 0.670 | | | | 35 | | | | 0.931 | | | |
|  | **1000** | | PFC | | 23.676 | | | 0.676 | | | | 35 | | | | 0.927 | | | |
|  |  |  | MNND | | 23.122 | | | 0.661 | | | | 35 | | | | 0.938 | | | |
|  | **1500** | | MNND | | 23.205 | | | 0.663 | | | | 35 | | | | 0.937 | | | |
|  |  |  | PFC | | 22.995 | | | 0.657 | | | | 35 | | | | 0.941 | | | |
|  |  |  | SFC3 | | 22.417 | | | 0.640 | | | | 35 | | | | 0.951 | | | |
| *Lophostoma silvicolum* | | | | | | | | | | | | | | | | | | | |
| **Season** | **Spatial Scale (m)** | | **Model structure** | | ***Sum of squared Pearson residuals*** | | | **Ratio**  **(SSQ residuals/rdf)** | | | **Residual df** | | | | **p-value** | | | | |
| **Dry Season** | **250** | | LVS | | 30.241 | | | 0.864 | | | 35 | | | | 0.697 | | | | |
|  |  |  | SFC3 | | 20.866 | | | 0.596 | | | 35 | | | | 0.972 | | | | |
|  |  |  | MNND | | 20.533 | | | 0.587 | | | 35 | | | | 0.975 | | | | |
|  |  |  | PFC | | 21.373 | | | 0.611 | | | 35 | | | | 0.966 | | | | |
|  |  |  | ED | | 16.872 | | | 0.482 | | | 35 | | | | 0.996 | | | | |
|  | **500** | | ED+PD+MNND+MSI | | 28.327 | | | 0.885 | | | 32 | | | | 0.653 | | | | |
|  |  |  | PD | | 17.334 | | | 0.495 | | | 35 | | | | 0.995 | | | | |
|  | **750** | | PD | | 16.374 | | | 0.468 | | | 35 | | | | 0.997 | | | | |
|  | **1000** | | PD | | 19.796 | | | 0.566 | | | 35 | | | | 0.982 | | | | |
|  | **1500** | | PD | | 21.157 | | | 0.604 | | | 35 | | | | 0.969 | | | | |
| **Wet Season** | **250** | | SFC1+SFC2+SFC3 | | 25.801 | | | 0.782 | | | 33 | | | | 0.810 | | | | |
|  |  |  | SFC2 | | 26.305 | | | 0.752 | | | 35 | | | | 0.855 | | | | |
|  |  |  | SFC3 | | 25.023 | | | 0.715 | | | 35 | | | | 0.894 | | | | |
|  |  |  | PFC | | 25.125 | | | 0.718 | | | 35 | | | | 0.891 | | | | |
|  |  |  | LVS | | 27.540 | | | 0.787 | | | 35 | | | | 0.811 | | | | |
|  |  |  | PFC+SFC1+SFC2+SFC3 | | 26.970 | | | 0.843 | | | 32 | | | | 0.719 | | | | |
|  | **500** | | MNND | | 35.173 | | | 1.005 | | | 35 | | | | 0.460 | | | | |
|  | **750** | | MNND | | 28.577 | | | 0.816 | | | 35 | | | | 0.770 | | | | |
|  | **1000** | | PD | | 26.770 | | | 0.765 | | | 35 | | | | 0.839 | | | | |
|  |  |  | SFC2 | | 27.715 | | | 0.792 | | | 35 | | | | 0.805 | | | | |
|  | **1500** | | PD | | 26.302 | | | 0.751 | | | 35 | | | | 0.855 | | | | |
|  |  |  | ED | | 28.664 | | | 0.819 | | | 35 | | | | 0.767 | | | | |
|  |  |  | SFC2 | | 24.352 | | | 0.696 | | | 35 | | | | 0.911 | | | | |
| *Mimon crenulatum* | | | | | | | | | | | | | | | | | | | |
| **Season** | **Spatial Scale (m)** | | **Model structure** | | ***Sum of squared Pearson residuals*** | | | **Ratio**  **(SSQ residuals/rdf)** | | | **Residual df** | | | | **p-value** | | | | |
| **Dry Season** | **250** | | SFC1 | | 26.434 | | | 0.755 | | | 35 | | | | 0.851 | | | | |
|  | **500** | | SFC3 | | 14.392 | | | 0.411 | | | 35 | | | | 0.999 | | | | |
|  |  |  | SFC1+SFC2+SFC3 | | 24.556 | | | 0.744 | | | 33 | | | | 0.855 | | | | |
|  |  |  | MNND | | 12.631 | | | 0.361 | | | 35 | | | | 1.000 | | | | |
|  |  |  | SFC1 | | 17.442 | | | 0.498 | | | 35 | | | | 0.994 | | | | |
|  |  |  | PFC | | 14.276 | | | 0.408 | | | 35 | | | | 0.999 | | | | |
|  |  |  | PD | | 13.913 | | | 0.398 | | | 35 | | | | 0.999 | | | | |
|  | **750** | | SFC3 | | 15.523 | | | 0.444 | | | 35 | | | | 0.998 | | | | |
|  |  |  | PFC | | 14.830 | | | 0.424 | | | 35 | | | | 0.999 | | | | |
|  | **1000** | | SFC3 | | 15.775 | | | 0.451 | | | 35 | | | | 0.998 | | | | |
|  |  |  | PFC | | 15.026 | | | 0.429 | | | 35 | | | | 0.999 | | | | |
|  | **1500** | | SFC3 | | 15.643 | | | 0.447 | | | 35 | | | | 0.998 | | | | |
|  |  |  | PFC | | 15.020 | | | 0.429 | | | 35 | | | | 0.999 | | | | |
| **Wet Season** | **250** | | SFC1+SFC2+SFC3 | | 25.724 | | | 0.780 | | | 33 | | | | 0.813 | | | | |
|  | **500** | | SFC2 | | 17.637 | | | 0.504 | | | 35 | | | | 0.994 | | | | |
|  | **750** | | SFC2 | | 16.216 | | | 0.463 | | | 35 | | | | 0.997 | | | | |
|  |  |  | ED+PD+MNND+MSI | | 16.675 | | | 0.521 | | | 32 | | | | 0.988 | | | | |
|  | **1000** | | SFC2 | | 17.049 | | | 0.487 | | | 35 | | | | 0.995 | | | | |
|  | **1500** | | SFC2 | | 15.044 | | | 0.430 | | | 35 | | | | 0.999 | | | | |
|  |  |  | MNND | | 15.823 | | | 0.452 | | | 35 | | | | 0.998 | | | | |
|  |  |  | PFC+SFC1+SFC2+SFC3 | | 18.543 | | | 0.579 | | | 32 | | | | 0.972 | | | | |
|  |  |  | ED | | 12.702 | | | 0.363 | | | 35 | | | | 1 | | | | |
|  |  |  | SFC1 | | 12.106 | | | 0.346 | | | 35 | | | | 1 | | | | |
| *Trachops cirrhosus* | | | | | | | | | | | | | | | | | | | |
| **Season** | **Spatial Scale (m)** | | **Model structure** | | ***Sum of squared Pearson residuals*** | | | **Ratio**  **(SSQ residuals/rdf)** | | | **Residual df** | | | | **p-value** | | | | |
| **Dry Season** | **250** | | SFC1 | | 11.835 | | | 0.338 | | | 35 | | | | 1 | | | | |
|  |  |  | LVS | | 16.130 | | | 0.461 | | | 35 | | | | 0.997 | | | | |
|  |  |  | MNND | | 14.316 | | | 0.409 | | | 35 | | | | 0.999 | | | | |
|  | **500** | | ED | | 16.877 | | | 0.482 | | | 35 | | | | 0.996 | | | | |
|  |  |  | LVS | | 16.130 | | | 0.461 | | | 35 | | | | 0.997 | | | | |
|  |  |  | MSI | | 11.534 | | | 0.330 | | | 35 | | | | 1 | | | | |
|  |  |  | MNND | | 14.658 | | | 0.419 | | | 35 | | | | 0.999 | | | | |
|  |  |  | SFC3 | | 14.465 | | | 0.413 | | | 35 | | | | 0.999 | | | | |
|  | **750** | | MSI | | 28.052 | | | 0.801 | | | 35 | | | | 0.791 | | | | |
|  | **1000** | | SFC3 | | 15.522 | | | 0.443 | | | 35 | | | | 0.998 | | | | |
|  |  |  | MSI | | 16.478 | | | 0.471 | | | 35 | | | | 0.997 | | | | |
|  |  |  | PFC | | 15.633 | | | 0.447 | | | 35 | | | | 0.998 | | | | |
|  |  |  | LVS | | 16.130 | | | 0.461 | | | 35 | | | | 0.997 | | | | |
|  | **1500** | | MSI | | 18.882 | | | 0.539 | | | 35 | | | | 0.988 | | | | |
|  |  |  | PFC | | 15.995 | | | 0.457 | | | 35 | | | | 0.998 | | | | |
|  |  |  | SFC3 | | 15.584 | | | 0.445 | | | 35 | | | | 0.998 | | | | |
| **Wet Season** | **250** | | SFC3 | | 16.555 | | | 0.473 | | | 35 | | | | 0.997 | | | | |
|  |  |  | PFC | | 16.070 | | | 0.459 | | | 35 | | | | 0.997 | | | | |
|  | **500** | | MNND | | 20.298 | | | 0.580 | | | 35 | | | | 0.978 | | | | |
|  | **750** | | MNND | | 19.061 | | | 0.545 | | | 35 | | | | 0.987 | | | | |
|  | **1000** | | MNND | | 19.502 | | | 0.557 | | | 35 | | | | 0.984 | | | | |
|  | **1500** | | ED | | 29.276 | | | 0.836 | | | 35 | | | | 0.740 | | | | |
| *Pteronotus parnellii* | | | | | | | | | | | | | | | | | | | |
| **Season** | | **Spatial Scale (m)** | **Model structure** | | ***Sum of squared Pearson residuals*** | | | | **Ratio**  **(SSQ residuals/rdf)** | | | **Residual df** | | | | **p-value** | | | |
| **Dry Season** | | **250** | SFC1 | | 25.896 | | | | 0.740 | | | 35 | | | | 0.868 | | | |
|  |  | **500** | PFC | | 25.955 | | | | 0.742 | | | 35 | | | | 0.866 | | | |
|  |  |  | SFC3 | | 26.289 | | | | 0.751 | | | 35 | | | | 0.856 | | | |
|  |  |  | SFC1 | | 26.416 | | | | 0.755 | | | 35 | | | | 0.851 | | | |
|  |  |  | MNND | | 29.700 | | | | 0.849 | | | 35 | | | | 0.722 | | | |
|  |  | **750** | SFC1 | | 26.931 | | | | 0.769 | | | 35 | | | | 0.834 | | | |
|  |  | **1000** | SFC1 | | 22.203 | | | | 0.634 | | | 35 | | | | 0.954 | | | |
|  |  |  | SFC1+SFC2+SFC3 | | 23.969 | | | | 0.726 | | | 33 | | | | 0.875 | | | |
|  |  |  | MNND | | 25.466 | | | | 0.728 | | | 35 | | | | 0.881 | | | |
|  |  |  | LVS | | 26.148 | | | | 0.747 | | | 35 | | | | 0.860 | | | |
|  |  | **1500** | MNND | | 28.819 | | | | 0.823 | | | 35 | | | | 0.760 | | | |
| **Wet Season** | | **250** | ED+PD+MNND+MSI | | 20.661 | | | | 0.646 | | | 32 | | | | 0.939 | | | |
|  |  | **500** | PFC | | 16.675 | | | | 0.476 | | | 35 | | | | 0.996 | | | |
|  |  |  | SFC3 | | 16.729 | | | | 0.478 | | | 35 | | | | 0.996 | | | |
|  |  | **750** | PFC | | 17.296 | | | | 0.494 | | | 35 | | | | 0.995 | | | |
|  |  |  | SFC3 | | 17.461 | | | | 0.499 | | | 35 | | | | 0.994 | | | |
|  |  | **1000** | PFC | | 17.487 | | | | 0.500 | | | 35 | | | | 0.994 | | | |
|  |  |  | SFC3 | | 17.575 | | | | 0.502 | | | 35 | | | | 0.994 | | | |
|  |  | **1500** | PFC | | 17.376 | | | | 0.496 | | | 35 | | | | 0.994 | | | |
|  |  |  | SFC3 | | 17.256 | | | | 0.493 | | | 35 | | | | 0.995 | | | |
|  |  |  | MNND | | 16.758 | | | | 0.479 | | | 35 | | | | 0.996 | | | |

**Table S4** Number of captures for each phyllostomid bat species and *Pteronotus parnellii* sampled in the BDFFP. Central Amazon. Brazil. Ensemble abbreviations: ANIM = animalivore; FRUG = frugivore; NECT = nectarivore; SANG = sanguivore. Species analysed are highlighted in bold.

|  |  |  |  | **Dry Season** | | | |  | **Wet Season** | | | |  |  |
| --- | --- | --- | --- | --- | --- | --- | --- | --- | --- | --- | --- | --- | --- | --- |
| **Taxon** |  | **Ensemble** |  | **Cont. Forest** | **Fragment** | **Edge** | **Matrix** |  | **Cont. Forest** | **Fragment** | **Edge** | **Matrix** |  | **Total** |
| **Phyllostomidae** |  |  |  |  |  |  |  |  |  |  |  |  |  |  |
| *Ametrida centurio* |  | FRUG |  | 0 | 0 | 4 | 2 |  | 0 | 0 | 0 | 1 |  | 7 |
| *Anoura caudifer* |  | NECT |  | 0 | 0 | 1 | 0 |  | 1 | 1 | 1 | 1 |  | 5 |
| ***Artibeus cinereus*** |  | FRUG |  | 8 | 4 | 6 | 7 |  | 5 | 3 | 1 | 2 |  | 36 |
| ***Artibeus concolor*** |  | FRUG |  | 3 | 7 | 19 | 36 |  | 1 | 1 | 1 | 1 |  | 69 |
| ***Artibeus gnomus*** |  | FRUG |  | 5 | 3 | 6 | 3 |  | 5 | 2 | 4 | 9 |  | 37 |
| ***Artibeus lituratus*** |  | FRUG |  | 14 | 4 | 4 | 21 |  | 10 | 1 | 0 | 2 |  | 56 |
| ***Artibeus obscurus*** |  | FRUG |  | 10 | 12 | 32 | 46 |  | 13 | 13 | 3 | 5 |  | 134 |
| *Artibeus planirostris* |  | FRUG |  | 4 | 5 | 1 | 1 |  | 6 | 3 | 0 | 2 |  | 22 |
| ***Carollia brevicauda*** |  | FRUG |  | 5 | 9 | 9 | 10 |  | 13 | 20 | 30 | 37 |  | 133 |
| *Carollia castanea* |  | FRUG |  | 0 | 3 | 0 | 0 |  |  |  |  |  |  | 3 |
| ***Carollia perspicillata*** |  | FRUG |  | 168 | 381 | 201 | 240 |  | 132 | 256 | 353 | 405 |  | 2136 |
| *Choeroniscus minor* |  | NECT |  | 0 | 4 | 0 | 0 |  | 1 | 2 | 0 | 0 |  | 7 |
| *Chrotopterus auritus* |  | ANIM |  | 1 | 2 | 0 | 0 |  | 2 | 0 | 0 | 0 |  | 5 |
| *Desmodus rotundus* |  | SANG |  | 4 | 1 | 0 | 1 |  | 3 | 1 | 1 | 0 |  | 11 |
| *Glossophaga soricina* |  | NECT |  | 0 | 3 | 1 | 0 |  | 2 | 2 | 0 | 0 |  | 8 |
| *Glyphonycteris daviesi* | | ANIM |  | 1 | 0 | 0 | 0 |  | 2 | 0 | 0 | 2 |  | 5 |
| *Glyphonycteris sylvestris* | | ANIM |  |  |  |  |  |  | 1 | 0 | 0 | 0 |  | 1 |
| *Lampronycteris brachyotis* | | ANIM |  |  |  |  |  |  | 0 | 1 | 0 | 0 |  | 1 |
| ***Lonchophylla thomasi*** | | NECT |  | 7 | 6 | 1 | 2 |  | 6 | 10 | 3 | 0 |  | 35 |
| *Lophostoma brasiliense* | | ANIM |  | 0 | 0 | 1 | 0 |  | 1 | 0 | 3 | 0 |  | 5 |
| *Lophostoma carrikeri* |  | ANIM |  | 1 | 1 | 2 | 0 |  | 0 | 0 | 0 | 1 |  | 5 |
| *Lophostoma schulzi* |  | ANIM |  | 2 | 1 | 0 | 0 |  | 2 | 1 | 1 | 2 |  | 9 |
| ***Lophostoma silvicolum*** | | ANIM |  | 23 | 7 | 3 | 3 |  | 29 | 7 | 11 | 7 |  | 90 |
| *Mesophylla macconnelli* | | FRUG |  | 12 | 2 | 1 | 1 |  | 4 | 3 | 1 | 0 |  | 24 |
| *Micronycteris hirsuta* |  | ANIM |  | 0 | 1 | 0 | 0 |  | 0 | 0 | 0 | 1 |  | 2 |
| *Micronycteris megalotis* | | ANIM |  | 1 | 1 | 0 | 1 |  | 0 | 0 | 1 | 0 |  | 4 |
| *Micronycteris microtis* | | ANIM |  | 2 | 1 | 0 | 1 |  | 3 | 2 | 2 | 4 |  | 15 |
| *Micronycteris schmidtorum* | | ANIM |  | 0 | 1 | 0 | 0 |  |  |  |  |  |  | 1 |
| ***Mimon crenulatum*** |  | ANIM |  | 20 | 6 | 7 | 7 |  | 6 | 10 | 20 | 16 |  | 92 |
| *Phylloderma stenops* |  | ANIM |  | 1 | 3 | 0 | 1 |  | 6 | 0 | 3 | 2 |  | 16 |
| *Phyllostomus discolor* | | NECT |  | 2 | 3 | 1 | 0 |  | 1 | 0 | 2 | 1 |  | 10 |
| ***Phyllostomus elongatus*** | | ANIM |  | 16 | 4 | 1 | 0 |  | 5 | 3 | 3 | 1 |  | 33 |
| *Phyllostomus hastatus* | | ANIM |  |  |  |  |  |  | 1 | 1 | 1 | 0 |  | 3 |
| *Platyrrhinus helleri* |  | FRUG |  | 0 | 0 | 2 | 0 |  | 0 | 0 | 0 | 1 |  | 3 |
| ***Rhinophylla pumilio*** |  | FRUG |  | 63 | 108 | 34 | 37 |  | 54 | 72 | 90 | 86 |  | 544 |
| *Sturnira tildae* |  | FRUG |  | 1 | 1 | 1 | 7 |  | 0 | 0 | 4 | 12 |  | 26 |
| ***Tonatia saurophila*** |  | ANIM |  | 9 | 10 | 2 | 1 |  | 23 | 15 | 4 | 3 |  | 67 |
| ***Trachops cirrhosus*** |  | ANIM |  | 27 | 7 | 4 | 2 |  | 44 | 23 | 4 | 14 |  | 125 |
| *Trinycteris nicefori* |  | ANIM |  | 2 | 0 | 0 | 0 |  | 2 | 2 | 2 | 2 |  | 10 |
| *Uroderma bilobatum* |  | FRUG |  | 0 | 1 | 2 | 0 |  | 0 | 0 | 0 | 2 |  | 5 |
| *Vampyressa pusilla* |  | FRUG |  |  |  |  |  |  | 0 | 0 | 1 | 0 |  | 1 |
| *Vampyriscus bidens* |  | FRUG |  | 3 | 3 | 0 | 1 |  | 7 | 2 | 1 | 2 |  | 19 |
| *Vampyriscus brocki* |  | FRUG |  | 0 | 1 | 0 | 0 |  | 0 | 1 | 1 | 0 |  | 3 |
| **Mormoopidae** |  |  |  |  |  |  |  |  |  |  |  |  |  |  |
| ***Pteronotus parnellii*** |  | ANIM |  | 47 | 40 | 11 | 16 |  | 71 | 13 | 36 | 38 |  | 272 |
|  |  |  |  |  |  |  |  |  |  |  |  |  |  |  |
| **Total Captures** |  |  |  | 462 | 646 | 357 | 447 |  | 462 | 471 | 588 | 662 |  | 4095 |

**Table S5** Results of likelihood ratio tests for differences in the abundance of each species between seasons (dry and wet) and habitat types (interior, edge and matrix). Significant (adjusted P < 0.05) results are highlighted in bold.

|  | **Interaction (Season x Habitat Type)** | | | **Season** | | | **Habitat Type** | | |
| --- | --- | --- | --- | --- | --- | --- | --- | --- | --- |
| **Species** | **Pr (>Chisq)** | **Chisq** | **Df** | **Pr (>Chisq)** | **Chisq** | **Df** | **Pr (>Chisq)** | **Chisq** | **Df** |
| *Artibeus cinereus* | **0.0051** | 20.239 | 7 | **0.0005** | 11.98 | 1 | 0.6498 | 1.64 | 3 |
| *Artibeus concolor* | **< 2.2e-16** | 134.76 | 7 | **< 2.2e-16** | 111.27 | 1 | **0.0022** | 14.55 | 3 |
| *Artibeus gnomus* | 0.3649 | 7.6459 | 7 | 0.4975 | 0.46 | 1 | 0.271 | 3.91 | 3 |
| *Artibeus lituratus* | **4.626e-11** | 62.568 | 7 | **4.735e-08** | 29.82 | 1 | **0.0036** | 13.55 | 3 |
| *Artibeus obscurus* | **< 2.2e-16** | 134.71 | 7 | **< 2.2e-16** | 76.88 | 1 | 0.9863 | 0.14 | 3 |
| *Carollia brevicauda* | **0.0009** | 24.628 | 7 | **0.0006** | 11.75 | 1 | **0.004** | 13.32 | 3 |
| *Carollia perspicillata* | **1.96e-10** | 59.431 | 7 | **3.719e-11** | 43.76 | 1 | **0.007** | 12.10 | 3 |
| *Lophostoma silvicolum* | 0.2218 | 9.4524 | 7 | 0.5401 | 0.38 | 1 | **0.0387** | 8.38 | 3 |
| *Lonchophylla thomasi* | 0.061 | 13.492 | 7 | 0.7867 | 0.07 | 1 | 0.0502 | 7.81 | 3 |
| *Mimon crenulatum* | 0.2097 | 9.644 | 7 | 0.6297 | 0.23 | 1 | 0.3915 | 3.00 | 3 |
| *Phyllostomus elongatus* | **0.0437** | 14.453 | 7 | **0.0266** | 4.92 | 1 | 0.0573 | 7.51 | 3 |
| *Pteronotus parnellii* | **0.0007** | 25.28 | 7 | 0.6546 | 0.20 | 1 | 0.2929 | 3.72 | 3 |
| *Rhinophylla pumilio* | **0.0068** | 19.498 | 7 | 0.2262 | 1.46 | 1 | **0.016** | 10.33 | 3 |
| *Trachops cirrhosus* | **0.0006** | 25.782 | 7 | **0.0068** | 7.32 | 1 | **0.0168** | 10.22 | 3 |
| *Tonatia saurophila* | **0.0263** | 15.871 | 7 | **0.0319** | 4.60 | 1 | **0.0362** | 8.53 | 3 |

**Table S6** Results of multiple pairwise comparisons of GLMMs testing for differences in abundance of eleven species across the two seasons (dry and wet) and across the four habitat types (continuous forest. fragment. edge and matrix). Significant (adjusted P < 0.05) results are highlighted in bold.

| **Wet vs Dry** | **Species** | | | |  | **Wet vs Dry** | **Species** | | | |
| --- | --- | --- | --- | --- | --- | --- | --- | --- | --- | --- |
|  | *Artibeus cinereus* | | | |  |  | *Phyllostomus elongatus* | | | |
|  | Estimate | Std. Error | z value | Pr(>\|z\|) |  |  | Estimate | Std. Error | z value | Pr(>\|z\|) |
| Cont. Forest | -0.7019 | 0.6124 | -1.146 | 0.942 |  | Cont. Forest | -1.262 | 0.5638 | -2.239 | 0.275 |
| Fragment | -0.3157 | 0.7638 | -0.413 | 1.000 |  | Fragment | -0.3125 | 0.7603 | -0.411 | 1.000 |
| Edge | -2.5690 | 1.0801 | -2.378 | 0.237 |  | Edge | 0.2856 | 1.1515 | 0.248 | 1.000 |
| Matrix | -2.0293 | 0.8018 | -2.531 | 0.170 |  | Matrix | 18.8891 | 1024.0003 | 0.018 | 1.000 |
|  | *Artibeus concolor* | | | |  |  | *Rhinophylla pumilio* | | | |
|  | Estimate | Std. Error | z value | Pr(>\|z\|) |  |  | Estimate | Std. Error | z value | Pr(>\|z\|) |
| Cont. Forest | -1.1126 | 1.1544 | -0.964 | 0.9737 |  | Cont. Forest | -0.1637 | 0.1846 | -0.887 | 0.9862 |
| Fragment | -1.9607 | 1.0692 | -1.834 | 0.5527 |  | Fragment | -0.4195 | 0.1537 | -2.730 | 0.1084 |
| Edge | -3.9641 | 1.0235 | -3.873 | **0.0023** |  | Edge | 0.1519 | 0.1993 | 0.762 | 0.9944 |
| Matrix | -4.2974 | 1.0157 | -4.231 | **< 0.001** |  | Matrix | 0.1014 | 0.1982 | 0.511 | 0.9996 |
|  | *Artibeus lituratus* | | | |  |  | *Trachops cirrhosus* | | | |
|  | Estimate | Std. Error | z value | Pr(>\|z\|) |  |  | Estimate | Std. Error | z value | Pr(>\|z\|) |
| Cont. Forest | -3.425e-01 | 4.173e-01 | -0.821 | 0.9881 |  | Cont. Forest | 0.4345 | 0.2467 | 1.761 | 0.6131 |
| Fragment | -1.423e+00 | 1.146e+00 | -1.241 | 0.8915 |  | Fragment | 1.1339 | 0.4340 | 2.612 | 0.1338 |
| Edge | -2.009e+01 | 7.787e+03 | -0.003 | 1.0000 |  | Edge | -0.8602 | 0.7095 | -1.212 | 0.9165 |
| Matrix | -3.153e+00 | 7.465e-01 | -4.224 | **< 0.001** |  | Matrix | 1.1423 | 0.7571 | 1.509 | 0.7781 |
|  | *Artibeus obscurus* | | | |  |  | *Tonatia saurophila* | | | |
|  | Estimate | Std. Error | z value | Pr(>\|z\|) |  |  | Estimate | Std. Error | z value | Pr(>\|z\|) |
| Cont. Forest | 0.2542 | 0.4206 | 0.604 | 0.9983 |  | Cont. Forest | 0.9323 | 0.3932 | 2.371 | 0.229 |
| Fragment | 0.0499 | 0.4006 | 0.125 | 1.0000 |  | Fragment | 0.4055 | 0.4083 | 0.993 | 0.971 |
| Edge | -3.2189 | 0.6027 | -5.341 | **< 0.001** |  | Edge | -0.0918 | 0.8682 | -0.106 | 1.000 |
| Matrix | -2.9699 | 0.4724 | -6.287 | **< 0.001** |  | Matrix | 0.2973 | 1.1567 | 0.257 | 1.000 |
|  | *Carollia brevicauda* | | | |  |  | *Pteronotus parnellii* | | | |
|  | Estimate | Std. Error | z value | Pr(>\|z\|) |  |  | Estimate | Std. Error | z value | Pr(>\|z\|) |
| Cont. Forest | 0.945 | 0.5082 | 1.860 | 0.5456 |  | Cont. Forest | 0.4322 | 0.1893 | 2.283 | 0.2805 |
| Fragment | 0.7836 | 0.3892 | 2.013 | 0.4401 |  | Fragment | -1.1431 | 0.3194 | -3.579 | **0.0076** |
| Edge | 0.3134 | 0.372 | 0.843 | 0.9886 |  | Edge | 0.3347 | 0.3462 | 0.967 | 0.9755 |
| Matrix | 0.5993 | 0.3618 | 1.656 | 0.6858 |  | Matrix | 0.1672 | 0.3062 | 0.546 | 0.9993 |
|  | *Carollia perspicillata* | | | |  |  |  |  |  |  |
|  | Estimate | Std. Error | z value | Pr(>\|z\|) |  |  |  |  |  |  |
| Cont. Forest | -0.2435 | 0.1156 | -2.106 | 0.3218 |  |  |  |  |  |  |
| Fragment | -0.3989 | 0.0811 | -4.919 | **<0.001** |  |  |  |  |  |  |
| Edge | -0.2696 | 0.088 | -3.065 | **0.0295** |  |  |  |  |  |  |
| Matrix | -0.2585 | 0.0833 | -3.105 | **0.0261** |  |  |  |  |  |  |

**Table S7** Results of model consistency between dry and wet season for bat-landscape relationships.

| **Species** | **Model Consistency (%)** |
| --- | --- |
| *Artibeus obscurus* | 0 |
| *Carollia brevicauda* | 71.43 |
| *Carollia perspicillata* | 71.43 |
| *Rhinophylla pumilio* | 25 |
| *Lophostoma silvicolum* | 40 |
| *Mimon crenulatum* | 28.57 |
| *Trachops cirrhosus* | 33.33 |
| *Pteronotus parnellii* | 37.50 |

**Table S8** Summary results of model averaging of the best-fit GLMMs (Akaike differences < 2 from the best model) investigating the relationship between local and landscape-scale attributes and the abundance of eight species for the wet and dry seasons and for five focal scales across the BDFFP. Central Amazon. Brazil. Predictor abbreviations: PFC - primary forest cover; SFC1 - initial secondary forest cover (≤ 5 years); SFC2 - intermediate secondary forest cover (6-15 years); SFC3 - advanced secondary forest cover (≥ 16 years); ED - edge density; PD - patch density; MNND - mean nearest neighbour distance; MSI - mean shape index.

| *Artibeus obscurus* | | | | **Local-scale** | **Compositional Predictors** | | | | **Configurational Predictors** | | | |
| --- | --- | --- | --- | --- | --- | --- | --- | --- | --- | --- | --- | --- |
| **Season** | **Spatial Scale (m)** | **Explanatory Variables** | **Intercept** | **Vegetation**  **structure** | **PFC** | **SFC1** | **SFC2** | **SFC3** | **PD** | **ED** | **MNND** | **MSI** |
| **Dry Season** | **250** | **Estimate (±SE)** | -6.16(0.69) | -0.85(0.33) |  | 0.93(0.41) | -0.56(0.6) | 0.61(0.48) | 0.15(0.61) | 0.32(0.56) | -1.4(0.5) | 2.11(0.51) |
|  |  | **95% Confidence interval** | -7.51 ; -4.81 | -1.5 ; -0.21 |  | 0.13 ; 1.74 | -1.73 ; 0.61 | -0.34 ; 1.56 | -1.04 ; 1.34 | -0.77 ; 1.41 | -2.37 ; -0.42 | 1.11 ; 3.12 |
|  |  | **Hierarchical Partitioning (%)** |  | 10.56 |  | 44.30 | 10.23 | 3.59 | 10.05 | 4.77 | 6.28 | 10.23 |
|  | **500** | **Estimate (±SE)** | -6.17(0.71) | -0.57(0.34) | -0.73(0.44) | 0.52(0.5) |  | 0.78(0.44) |  |  |  |  |
|  |  | **95% Confidence interval** | -7.57 ; -4.78 | -1.24 ; 0.09 | -1.6 ; 0.14 | -0.46 ; 1.51 |  | -0.08 ; 1.64 |  |  |  |  |
|  |  | **Hierarchical Partitioning (%)** |  | 17.31 | 26.00 | 21.34 |  | 35.35 |  |  |  |  |
|  | **750** | **Estimate (±SE)** | -6.09(0.66) | -0.57(0.34) | -0.79(0.46) |  |  | 0.93(0.43) |  |  |  |  |
|  |  | **95% Confidence interval** | -7.38 ; -4.79 | -1.24 ; 0.09 | -1.69 ; 0.12 |  |  | 0.09 ; 1.77 |  |  |  |  |
|  |  | **Hierarchical Partitioning (%)** |  | 9.62 | 31.80 |  |  | 58.58 |  |  |  |  |
|  | **1000** | **Estimate (±SE)** | -6.13(0.66) | -0.57(0.34) | -0.73(0.47) | -0.62(0.48) | -0.53(0.35) | 1.04(0.41) | -0.83(0.54) |  |  |  |
|  |  | **95% Confidence interval** | -7.42 ; -4.84 | -1.24 ; 0.09 | -1.66 ; 0.2 | -1.55 ; 0.32 | -1.22 ; 0.17 | 0.23 ; 1.85 | -1.89 ; 0.23 |  |  |  |
|  |  | **Hierarchical Partitioning (%)** |  | 7.74 | 20.67 | 2.71 | 22.16 | 28.37 | 18.35 |  |  |  |
|  | **1500** | **Estimate (±SE)** | -6.01(0.39) |  |  | -0.59(0.54) | -0.79(0.42) | 1.26(0.32) |  |  |  |  |
|  |  | **95% Confidence interval** | -6.77 ; -5.26 |  |  | -1.64 ; 0.47 | -1.6 ; 0.03 | 0.63 ; 1.89 |  |  |  |  |
|  |  | **Hierarchical Partitioning (%)** |  |  |  | 7.20 | 37.25 | 55.56 |  |  |  |  |
| **Wet Season** | **250** | **Estimate (±SE)** | -6.23(0.32) | 0.49 (0.27) | 0.41(0.21) | -0.46(0.29) | -0.53(0.27) | -0.32(0.22) |  |  |  |  |
|  |  | **95% Confidence interval** | -6.85 ; -5.61 | -0.03 ; 1.02 | -0.01 ; 0.83 | -1.02 ; 0.1 | -1.06 ; 0.01 | -0.75 ; 0.1 |  |  |  |  |
|  |  | **Hierarchical Partitioning (%)** |  | 23.84 | 26.97 | 12.11 | 19.03 | 18.05 |  |  |  |  |
|  | **500** | **Estimate (±SE)** | -6.23(0.33) | 0.49(0.27) | 0.37(0.22) |  |  | -0.38(0.22) | -0.71(0.36) |  |  |  |
|  |  | **95% Confidence interval** | -6.87 ; -5.59 | -0.03 ; 1.02 | -0.06 ; 0.79 |  |  | -0.81 ; 0.05 | -1.4 ; -0.01 |  |  |  |
|  |  | **Hierarchical Partitioning (%)** |  | 32.59 | 19.80 |  |  | 23.86 | 23.75 |  |  |  |
|  | **750** | **Estimate (±SE)** | -6.24(0.32) | 0.49(0.27) | 0.29(0.22) |  |  | -0.3(0.23) |  |  | -0.33(0.23) |  |
|  |  | **95% Confidence interval** | -6.87 ; -5.6 | -0.03 ; 1.02 | -0.14 ; 0.73 |  |  | -0.74 ; 0.15 |  |  | -0.78 ; 0.13 |  |
|  |  | **Hierarchical Partitioning (%)** |  | 48.01 | 14.45 |  |  | 15.28 |  |  | 22.27 |  |
|  | **1000** | **Estimate (±SE)** | -6.25(0.32) | 0.49(0.27) |  |  |  |  |  |  | -0.32(0.23) |  |
|  |  | **95% Confidence interval** | -6.88 ; -5.62 | -0.03 ; 1.02 |  |  |  |  |  |  | -0.77 ; 0.13 |  |
|  |  | **Hierarchical Partitioning (%)** |  | 66.33 |  |  |  |  |  |  | 33.67 |  |
|  | **1500** | **Estimate (±SE)** | -6.25(0.33) | 0.49(0.27) |  |  | -0.46(0.26) |  |  |  | -0.34(0.22) |  |
|  |  | **95% Confidence interval** | -6.88 ; -5.61 | -0.03 ; 1.02 |  |  | -0.96 ; 0.05 |  |  |  | -0.77 ; 0.1 |  |
|  |  | **Hierarchical Partitioning (%)** |  | 43.00 |  |  | 31.92 |  |  |  | 25.08 |  |
| *Carollia brevicauda* | | | | **Local-scale** | **Compositional Predictors** | | | | **Configurational Predictors** | | | |
| **Season** | **Spatial Scale (m)** | **Explanatory Variables** | **Intercept** | **Vegetation**  **structure** | **FC** | **SF 1** | **SF 2** | **SF 3** | **PD** | **ED** | **MNND** | **MSI** |
| **Dry Season** | **250** | **Estimate (±SE)** | -5.86(0.44) | -0.67(0.27) | -0.7(0.28) |  |  | 0.64(0.27) | -0.11(0.27) | 0.68(0.34) | -0.27(0.35) | 0.02(0.32) |
|  |  | **95% Confidence interval** | -6.73 ; -4.99 | -1.19 ; -0.14 | -1.24 ; -0.16 |  |  | 0.11 ; 1.16 | -0.65 ; 0.42 | 0.01 ; 1.36 | -0.97 ; 0.42 | -0.6 ; 0.65 |
|  |  | **Hierarchical Partitioning (%)** |  | 1.56 | 12.25 |  |  | 5.67 | 8.81 | 48.67 | 9.88 | 13.17 |
|  | **500** | **Estimate (±SE)** | -5.82(0.41) | -0.56(0.23) | -0.69(0.29) |  |  | 0.64(0.28) |  |  |  |  |
|  |  | **95% Confidence interval** | -6.63 ; -5.01 | -1.01 ; -0.11 | -1.27 ; -0.11 |  |  | 0.09 ; 1.2 |  |  |  |  |
|  |  | **Hierarchical Partitioning (%)** |  | 6.66 | 30.84 |  |  | 62.50 |  |  |  |  |
|  | **750** | **Estimate (±SE)** | -5.82(0.4) | -0.56(0.23) | -0.66(0.33) |  |  | 0.66(0.27) |  |  |  |  |
|  |  | **95% Confidence interval** | -6.62 ; -5.03 | -1.01 ; -0.11 | -1.3 ; -0.02 |  |  | 0.13 ; 1.19 |  |  |  |  |
|  |  | **Hierarchical Partitioning (%)** |  | 3.27 | 33.22 |  |  | 63.52 |  |  |  |  |
|  | **1000** | **Estimate (±SE)** | -5.84(0.36) | -0.5(0.26) | -0.55(0.21) |  |  | 0.6(0.26) | -0.78(0.53) | -0.42(0.48) | 0.04(0.42) | 0.21(0.38) |
|  |  | **95% Confidence interval** | -6.54 ; -5.15 | -1.01 ; 0 | -0.96 ; -0.14 |  |  | 0.09 ; 1.11 | -1.82 ; 0.25 | -1.35 ; 0.51 | -0.79 ; 0.87 | -0.54 ; 0.96 |
|  |  | **Hierarchical Partitioning (%)** |  | 2.18 | 16.35 |  |  | 24.61 | 22.99 | 20.07 | 10.77 | 3.04 |
|  | **1500** | **Estimate (±SE)** | -5.93(0.27) | -0.38(0.22) |  |  |  |  | -0.2(0.46) | -0.75(0.32) | 0.98(0.24) | 0.44(0.23) |
|  |  | **95% Confidence interval** | -6.45 ; -5.4 | -0.8 ; 0.05 |  |  |  |  | -1.09 ; 0.69 | -1.37 ; -0.13 | 0.51 ; 1.45 | -0.01 ; 0.9 |
|  |  | **Hierarchical Partitioning (%)** |  | 3.73 |  |  |  |  | 14.94 | 33.63 | 29.75 | 17.95 |
| **Wet Season** | **250** | **Estimate (±SE)** | -5.01(0.22) | -0.44(0.16) | -0.47(0.2) |  |  | 0.48(0.18) |  |  |  |  |
|  |  | **95% Confidence interval** | -5.44 ; -4.57 | -0.75 ; -0.12 | -0.87 ; -0.07 |  |  | 0.14 ; 0.83 |  |  |  |  |
|  |  | **Hierarchical Partitioning (%)** |  | 20.28 | 30.32 |  |  | 49.40 |  |  |  |  |
|  | **500** | **Estimate (±SE)** | -5.01(0.23) | -0.44(0.16) | -0.51(0.24) |  |  | 0.49(0.22) |  |  |  | 0.38(0.18) |
|  |  | **95% Confidence interval** | -5.47 ; -4.55 | -0.75 ; -0.12 | -0.98 ; -0.03 |  |  | 0.06 ; 0.91 |  |  |  | 0.02 ; 0.74 |
|  |  | **Hierarchical Partitioning (%)** |  | 27.6 | 14.08 |  |  | 20.13 |  |  |  | 38.20 |
|  | **750** | **Estimate (±SE)** | -4.95(0.29) | -0.38(0.17) | -0.78(0.28) |  |  |  | -1.27(0.38) | 0.67(0.3) | 0.2(0.19) | -0.38(0.24) |
|  |  | **95% Confidence interval** | -5.53 ; -4.37 | -0.72 ; -0.05 | -1.34 ; -0.23 |  |  |  | -2.02 ; -0.52 | 0.08 ; 1.27 | -0.17 ; 0.56 | -0.84 ; 0.09 |
|  |  | **Hierarchical Partitioning (%)** |  | 20.10 | 6.01 |  |  |  | 34.72 | 16.16 | 12.77 | 10.24 |
|  | **1000** | **Estimate (±SE)** | -5(0.23) | -0.44(0.16) |  |  |  |  |  |  |  |  |
|  |  | **95% Confidence interval** | -5.44 ; -4.56 | -0.75 ; -0.12 |  |  |  |  |  |  |  |  |
|  |  | **Hierarchical Partitioning (%)** |  | 100 |  |  |  |  |  |  |  |  |
|  | **1500** | **Estimate (±SE)** | -4.96(0.28) | -0.44(0.16) |  |  |  |  |  |  | 0.67(0.26) |  |
|  |  | **95% Confidence interval** | -5.51 ; -4.41 | -0.75 ; -0.12 |  |  |  |  |  |  | 0.16 ; 1.18 |  |
|  |  | **Hierarchical Partitioning (%)** |  | 63.68 |  |  |  |  |  |  | 36.32 |  |
| *Carollia perspicillata* | | | | **Local-scale** | **Compositional Predictors** | | | | **Configurational Predictors** | | | |
| **Season** | **Spatial Scale (m)** | **Explanatory Variables** | **Intercept** | **Vegetation**  **structure** | **FC** | **SF 1** | **SF 2** | **SF 3** | **PD** | **ED** | **MNND** | **MSI** |
| **Dry Season** | **250** | **Estimate (±SE)** | -2.27(0.17) | -0.27(0.13) | -0.38(0.19) |  |  |  | 0.05(0.15) | 0.45(0.17) | 0.05(0.2) | -0.21(0.21) |
|  |  | **95% Confidence interval** | -2.61 ; -1.93 | -0.52 ; -0.02 | -0.76 ; 0 |  |  |  | -0.24 ; 0.34 | 0.11 ; 0.79 | -0.33 ; 0.44 | -0.62 ; 0.21 |
|  |  | **Hierarchical Partitioning (%)** |  | 8.22 | 6.15 |  |  |  | 10.96 | 65.29 | 3.03 | 6.36 |
|  | **500** | **Estimate (±SE)** | -2.24(0.14) | -0.19(0.11) | -0.69(0.21) |  |  | 0.58(0.11) | -0.28(0.17) | 0.15(0.12) | -0.42(0.17) | -0.1(0.12) |
|  |  | **95% Confidence interval** | -2.51 ; -1.97 | -0.41 ; 0.03 | -1.1 ; -0.29 |  |  | 0.36 ; 0.79 | -0.61 ; 0.05 | -0.08 ; 0.38 | -0.75 ; -0.09 | -0.34 ; 0.14 |
|  |  | **Hierarchical Partitioning (%)** |  | 15.83 | 29.01 |  |  | 16.88 | 7.14 | 2.58 | 23.12 | 5.45 |
|  | **750** | **Estimate (±SE)** | -2.23(0.13) |  | -0.6(0.13) |  |  | 0.62(0.11 ) |  |  |  |  |
|  |  | **95% Confidence interval** | -2.49 ; -1.98 |  | -0.86 ; -0.35 |  |  | 0.4 ; 0.84 |  |  |  |  |
|  |  | **Hierarchical Partitioning (%)** |  |  | 36.73 |  |  | 63.27 |  |  |  |  |
|  | **1000** | **Estimate (±SE)** | -2.25(0.14) | -0.12(0.12) | -0.7(0.16) |  |  | 0.61(0.12) | -0.46(0.25) | -0.29(0.21) | 0.11(0.16) | 0.12(0.19) |
|  |  | **95% Confidence interval** | -2.52 ; -1.97 | -0.34 ; 0.11 | -1.02 ; -0.38 |  |  | 0.38 ; 0.84 | -0.94 ; 0.02 | -0.7 ; 0.11 | -0.21 ; 0.42 | -0.25 ; 0.5 |
|  |  | **Hierarchical Partitioning (%)** |  | 14.07 | 25.70 |  |  | 33.76 | 7.39 | 3.78 | 11.23 | 4.07 |
|  | **1500** | **Estimate (±SE)** | -2.28(0.26) | -0.08(0.09) | -0.88(0.23) |  |  |  | -0.92(0.28) | -0.25(0.2) | 0.34(0.15) | -0.01(0.13) |
|  |  | **95% Confidence interval** | -2.78 ; -1.78 | -0.26 ; 0.1 | -1.32 ; -0.44 |  |  |  | -1.48 ; -0.36 | -0.65 ; 0.15 | 0.05 ; 0.64 | -0.25 ; 0.24 |
|  |  | **Hierarchical Partitioning (%)** |  | 21.50 | 40.61 |  |  |  | 12.55 | 6.57 | 13.28 | 5.49 |
| **Wet Season** | **250** | **Estimate (±SE)** | -2.47(0.12) | -0.38(0.11) |  |  |  |  | 0.03(0.16) | 0.34(0.15) | 0.16(0.15) | -0.03(0.17) |
|  |  | **95% Confidence interval** | -2.7 ; -2.24 | -0.6 ; -0.17 |  |  |  |  | -0.3 ; 0.35 | 0.05 ; 0.63 | -0.14 ; 0.45 | -0.36 ; 0.3 |
|  |  | **Hierarchical Partitioning (%)** |  | 34.68 |  |  |  |  | 16.33 | 33.77 | 10.31 | 4.91 |
|  | **500** | **Estimate (±SE)** | -2.47(0.08) | -0.29(0.1) | -0.55(0.15) |  |  |  | -0.72(0.16) | 0.49(0.11) | -0.07(0.16) | -0.38(0.12) |
|  |  | **95% Confidence interval** | -2.62 ; -2.32 | -0.5 ; -0.09 | -0.84 ; -0.27 |  |  |  | -1.04 ; -0.4 | 0.26 ; 0.71 | -0.37 ; 0.24 | -0.61 ; -0.16 |
|  |  | **Hierarchical Partitioning (%)** |  | 14.99 | 38.44 |  |  |  | 16.23 | 11.29 | 10.23 | 8.82 |
|  | **750** | **Estimate (±SE)** | -2.47(0.08) | -0.33(0.11) | -0.42(0.12) |  |  |  | -0.65(0.18) | 0.61(0.16) | -0.21(0.13) | -0.5(0.13) |
|  |  | **95% Confidence interval** | -2.63 ; -2.32 | -0.54 ; -0.11 | -0.66 ; -0.18 |  |  |  | -1 ; -0.3 | 0.3 ; 0.92 | -0.47 ; 0.06 | -0.76 ; -0.24 |
|  |  | **Hierarchical Partitioning (%)** |  | 16.4 | 49.36 |  |  |  | 8.13 | 4.53 | 11.64 | 9.94 |
|  | **1000** | **Estimate (±SE)** | -2.48(0.14) | -0.33(0.12) | -0.64(0.22) |  |  | 0.51(0.11) | -0.95(0.3) | 0.38(0.22) | -0.31(0.17) | -0.45(0.22) |
|  |  | **95% Confidence interval** | -2.76 ; -2.2 | -0.56 ;-0.11 | -1.07 ;-0.2 |  |  | 0.3 ;0.72 | -1.54 ;-0.37 | -0.04 ;0.8 | -0.64 ;0.02 | -0.88 ; -0.01 |
|  |  | **Hierarchical Partitioning (%)** |  | 12.11 | 35.86 |  |  | 31.48 | 8.42 | 2.69 | 8.30 | 1.14 |
|  | **1500** | **Estimate (±SE)** | -2.48(0.1) |  |  |  |  | 0.53(0.11) |  |  |  |  |
|  |  | **95% Confidence interval** | -2.68 ;-2.27 |  |  |  |  | 0.32 ;0.74 |  |  |  |  |
|  |  | **Hierarchical Partitioning (%)** |  |  |  |  |  | 100 |  |  |  |  |
| *Rhinophylla pumilio* | | | | **Local-scale** | **Compositional Predictors** | | | | **Configurational Predictors** | | | |
| **Season** | **Spatial Scale (m)** | **Explanatory Variables** | **Intercept** | **Vegetation**  **structure** | **FC** | **SF 1** | **SF 2** | **SF 3** | **PD** | **ED** | **MNND** | **MSI** |
| **Dry Season** | **250** | **Estimate (±SE)** | -3.67(0.19) |  |  | -0.2(0.08) |  |  |  | 0.17(0.07) |  |  |
|  |  | **95% Confidence interval** | -4.05 ;-3.29 |  |  | -0.36 ;-0.04 |  |  |  | 0.02 ;0.31 |  |  |
|  |  | **Hierarchical Partitioning (%)** |  |  |  | 11.13 |  |  |  | 88.87 |  |  |
|  | **500** | **Estimate (±SE)** | -3.65(0.18) | 0.04(0.1) | -0.05(0.11) | -0.03(0.12) | 0.09(0.1) | 0.05(0.12) | -0.12(0.12) | 0.09(0.11) | 0(0.11) | -0.03(0.09) |
|  |  | **95% Confidence interval** | -4 ;-3.31 | -0.15 ;0.23 | -0.27 ;0.16 | -0.26 ;0.19 | -0.11 ;0.29 | -0.18 ;0.29 | -0.36 ;0.11 | -0.12 ;0.3 | -0.21 ;0.2 | -0.2 ; 0.14 |
|  |  | **Hierarchical Partitioning (%)** |  | 23.23 | 7.26 | 3.44 | 5.99 | 5.98 | 37.14 | 7.47 | 2.41 | 7.10 |
|  | **750** | **Estimate (±SE)** | -3.61(0.12) | 0.06(0.09) | -0.38(0.08) |  |  |  | -0.63(0.34) | 0.33(0.12) | 0.05(0.12) | -0.32(0.1) |
|  |  | **95% Confidence interval** | -3.85 ;-3.37 | -0.12 ;0.24 | -0.54 ;-0.22 |  |  |  | -1.3 ;0.04 | 0.1 ;0.57 | -0.2 ;0.29 | -0.51 ; -0.12 |
|  |  | **Hierarchical Partitioning (%)** |  | 35.17 | 19.35 |  |  |  | 14.91 | 4.31 | 20.32 | 5.95 |
|  | **1000** | **Estimate (±SE)** | -3.64(0.17) | 0.04(0.1) | -0.15(0.11) | 0.11(0.17) | 0.05(0.1) | 0.17(0.12) | -0.11(0.12) | 0.09(0.14) | -0.05(0.11) | 0.09(0.09) |
|  |  | **95% Confidence interval** | -3.98 ;-3.3 | -0.15 ;0.23 | -0.37 ;0.07 | -0.22 ;0.44 | -0.15 ;0.24 | -0.06 ;0.39 | -0.35 ;0.14 | -0.18 ;0.36 | -0.26 ;0.16 | -0.09 ; 0.27 |
|  |  | **Hierarchical Partitioning (%)** |  | 26.41 | 11.83 | 2.05 | 5.23 | 11.69 | 8.39 | 2.35 | 31.09 | 0.96 |
|  | **1500** | **Estimate (±SE)** | -3.6(0.16) |  | -0.18(0.11) | 0.16(0.12) |  | 0.2(0.11) |  |  | 0.13(0.11) |  |
|  |  | **95% Confidence interval** | -3.91 ;-3.29 |  | -0.41 ;0.04 | -0.08 ;0.41 |  | -0.03 ;0.42 |  |  | -0.08 ;0.35 |  |
|  |  | **Hierarchical Partitioning (%)** |  |  | 24.18 | 13.54 |  | 35.08 |  |  | 27.20 |  |
| **Wet Season** | **250** | **Estimate (±SE)** | -3.62(0.08) | -0.2(0.08) | -0.22(0.08) | -0.14(0.09) | 0.07(0.08) | 0.23(0.09) |  |  |  |  |
|  |  | **95% Confidence interval** | -3.77 ;-3.46 | -0.36 ;-0.05 | -0.38 ;-0.06 | -0.31 ;0.03 | -0.09 ;0.23 | 0.07 ;0.4 |  |  |  |  |
|  |  | **Hierarchical Partitioning (%)** |  | 7.98 | 19.45 | 30.21 | 21.62 | 20.73 |  |  |  |  |
|  | **500** | **Estimate (±SE)** | -3.62(0.08) | -0.2(0.08) | -0.22(0.08) |  | 0.22(0.08) |  |  | 0.21(0.08) |  |  |
|  |  | **95% Confidence interval** | -3.78 ;-3.46 | -0.36 ;-0.05 | -0.38 ;-0.06 |  | 0.07 ;0.38 |  |  | 0.05 ;0.37 |  |  |
|  |  | **Hierarchical Partitioning (%)** |  | 10.38 | 17.04 |  | 35.16 |  |  | 37.42 |  |  |
|  | **750** | **Estimate (±SE)** | -3.62(0.08) |  | -0.24(0.08) |  |  |  |  |  |  |  |
|  |  | **95% Confidence interval** | -3.77 ;-3.47 |  | -0.4 ;-0.09 |  |  |  |  |  |  |  |
|  |  | **Hierarchical Partitioning (%)** |  |  | 100 |  |  |  |  |  |  |  |
|  | **1000** | **Estimate (±SE)** | -3.62(0.08) |  | -0.25(0.08) |  |  |  |  |  | 0.23(0.08) |  |
|  |  | **95% Confidence interval** | -3.77 ;-3.47 |  | -0.4 ;-0.1 |  |  |  |  |  | 0.08 ;0.38 |  |
|  |  | **Hierarchical Partitioning (%)** |  |  | 73.70 |  |  |  |  |  | 26.30 |  |
|  | **1500** | **Estimate (±SE)** | -3.62(0.08) |  | -0.23(0.08) |  |  | 0.21(0.08) |  |  | 0.24(0.08) |  |
|  |  | **95% Confidence interval** | -3.77 ;-3.46 |  | -0.39 ;-0.08 |  |  | 0.06 ;0.37 |  |  | 0.08 ;0.4 |  |
|  |  | **Hierarchical Partitioning (%)** |  |  | 50.26 |  |  | 31.08 |  |  | 18.66 |  |
| *Lophostoma silvicolum* | | | | **Local-scale** | **Compositional Predictors** | | | | **Configurational Predictors** | | | |
| **Season** | **Spatial Scale (m)** | **Explanatory Variables** | **Intercept** | **Vegetation**  **structure** | **FC** | **SF 1** | **SF 2** | **SF 3** | **PD** | **ED** | **MNND** | **MSI** |
| **Dry Season** | **250** | **Estimate (±SE)** | -5.76(0.3) | 0.47(0.3) | 0.28(0.2) |  |  | -0.36(0.22) |  | 0.26(0.25) | -0.31(0.28) |  |
|  |  | **95% Confidence interval** | -6.35 ;-5.17 | -0.12 ;1.07 | -0.11 ;0.66 |  |  | -0.78 ;0.06 |  | -0.23 ;0.76 | -0.86 ;0.23 |  |
|  |  | **Hierarchical Partitioning (%)** |  | 39.63 | 20.98 |  |  | 25.40 |  | 1.59 | 12.40 |  |
|  | **500** | **Estimate (±SE)** | -5.96(0.29) |  |  |  |  |  | 0.14(0.8) | -0.45(0.26) | -1.11(0.37) | 0.89(0.27) |
|  |  | **95% Confidence interval** | -6.52 ;-5.39 |  |  |  |  |  | -1.43 ;1.71 | -0.97 ;0.06 | -1.84 ;-0.39 | 0.35 ; 1.42 |
|  |  | **Hierarchical Partitioning (%)** |  |  |  |  |  |  | 18.76 | 11.70 | 61.32 | 8.22 |
|  | **750** | **Estimate (±SE)** | -5.95(0.31) |  |  |  |  |  | -0.89(0.33) |  |  |  |
|  |  | **95% Confidence interval** | -6.56 ;-5.33 |  |  |  |  |  | -1.54 ;-0.24 |  |  |  |
|  |  | **Hierarchical Partitioning (%)** |  |  |  |  |  |  | 100 |  |  |  |
|  | **1000** | **Estimate (±SE)** | -6.03(0.33) |  |  |  |  |  | -1.08(0.36) |  |  |  |
|  |  | **95% Confidence interval** | -6.68 ;-5.38 |  |  |  |  |  | -1.79 ;-0.37 |  |  |  |
|  |  | **Hierarchical Partitioning (%)** |  |  |  |  |  |  | 100 |  |  |  |
|  | **1500** | **Estimate (±SE)** | -5.95(0.3) |  |  |  |  |  | -0.85(0.28) |  |  |  |
|  |  | **95% Confidence interval** | -6.53 ;-5.37 |  |  |  |  |  | -1.4 ;-0.31 |  |  |  |
|  |  | **Hierarchical Partitioning (%)** |  |  |  |  |  |  | 100 |  |  |  |
| **Wet Season** | **250** | **Estimate (±SE)*** | -5.63(0.22) | 0.35(0.19) | 17.47(32.75) | 2.72(5.29) | 2.08(6.99) | 5.31(17.65) |  |  |  |  |
|  |  | **95% Confidence interval*** | -6.07 ;-5.19 | -0.03 ;0.72 | -46.71 ;81.65 | -7.65 ;13.09 | -11.63 ;15.78 | -29.28 ;39.9 |  |  |  |  |
|  |  | **Hierarchical Partitioning (%)** |  | 20.91 | 24.18 | 12.12 | 18.13 | 24.66 |  |  |  |  |
|  | **500** | **Estimate (±SE)** | -5.64(0.21) |  |  |  |  |  |  |  | -0.6(0.17) |  |
|  |  | **95% Confidence interval** | -6.05 ;-5.24 |  |  |  |  |  |  |  | -0.94 ;-0.27 |  |
|  |  | **Hierarchical Partitioning (%)** |  |  |  |  |  |  |  |  | 100 |  |
|  | **750** | **Estimate (±SE)** | -5.67(0.21) |  |  |  |  |  |  |  | -0.63(0.18) |  |
|  |  | **95% Confidence interval** | -6.09 ;-5.26 |  |  |  |  |  |  |  | -0.98 ;-0.29 |  |
|  |  | **Hierarchical Partitioning (%)** |  |  |  |  |  |  |  |  | 100 |  |
|  | **1000** | **Estimate (±SE)** | -5.64(0.21) |  |  |  | -0.55(0.16) |  | -0.7(0.22) |  |  |  |
|  |  | **95% Confidence interval** | -6.06 ;-5.22 |  |  |  | -0.87 ;-0.24 |  | -1.14 ;-0.26 |  |  |  |
|  |  | **Hierarchical Partitioning (%)** |  |  |  |  | 62.45 |  | 37.55 |  |  |  |
|  | **1500** | **Estimate (±SE)** | -5.62(0.21) |  |  |  | -0.46(0.17) |  | -0.58(0.2) | -0.51(0.18) |  |  |
|  |  | **95% Confidence interval** | -6.04 ;-5.2 |  |  |  | -0.79 ;-0.14 |  | -0.97 ;-0.18 | -0.85 ;-0.16 |  |  |
|  |  | **Hierarchical Partitioning (%)** |  |  |  |  | 33.95 |  | 33.36 | 32.69 |  |  |
| *Mimon crenulatum* | | | | **Local-scale** | **Compositional Predictors** | | | | **Configurational Predictors** | | | |
| **Season** | **Spatial Scale (m)** | **Explanatory Variables** | **Intercept** | **Vegetation**  **structure** | **FC** | **SF 1** | **SF 2** | **SF 3** | **PD** | **ED** | **MNND** | **MSI** |
| **Dry Season** | **250** | **Estimate (±SE)** | -5.95(0.35) |  |  | -0.86(0.38) |  |  |  |  |  |  |
|  |  | **95% Confidence interval** | -6.63 ;-5.27 |  |  | -1.6 ;-0.13 |  |  |  |  |  |  |
|  |  | **Hierarchical Partitioning (%)** |  |  |  | 100 |  |  |  |  |  |  |
|  | **500** | **Estimate (±SE)** | -5.95(0.38) |  | 0.44(0.27) | -0.41(0.31) | 0.57(0.25) | -0.59(0.32) | -0.55(0.38) |  | -0.59(0.35) |  |
|  |  | **95% Confidence interval** | -6.7 ;-5.2 |  | -0.09 ;0.98 | -1.01 ;0.19 | 0.07 ;1.07 | -1.22 ;0.03 | -1.29 ;0.2 |  | -1.28 ;0.09 |  |
|  |  | **Hierarchical Partitioning (%)** |  |  | 26.70 | 7.42 | 6.56 | 31.21 | 5.39 |  | 22.72 |  |
|  | **750** | **Estimate (±SE)** | -5.93(0.36) |  | 0.43(0.26 ) |  |  | -0.49(0.27) |  |  |  |  |
|  |  | **95% Confidence interval** | -6.64 ;-5.22 |  | -0.09 ;0.94 |  |  | -1.02 ;0.05 |  |  |  |  |
|  |  | **Hierarchical Partitioning (%)** |  |  | 48.66 |  |  | 51.34 |  |  |  |  |
|  | **1000** | **Estimate (±SE)** | -5.91(0.36) |  | 0.41(0.26) |  |  | -0.47(0.27) |  |  |  |  |
|  |  | **95% Confidence interval** | -6.61 ;-5.21 |  | -0.1 ;0.91 |  |  | -0.99 ;0.06 |  |  |  |  |
|  |  | **Hierarchical Partitioning (%)** |  |  | 48.25 |  |  | 51.76 |  |  |  |  |
|  | **1500** | **Estimate (±SE)** | -5.92(0.36) |  | 0.47(0.27) |  |  | -0.5(0.27) |  |  |  |  |
|  |  | **95% Confidence interval** | -6.62 ;-5.22 |  | -0.06 ;0.99 |  |  | -1.03 ;0.03 |  |  |  |  |
|  |  | **Hierarchical Partitioning (%)** |  |  | 48.89 |  |  | 51.11 |  |  |  |  |
| **Wet Season** | **250** | **Estimate (±SE)** | -5.87(0.25) |  |  | -1.09(0.3) | 0.42(0.16) | 0.36(0.19) |  |  |  |  |
|  |  | **95% Confidence interval** | -6.35 ;-5.39 |  |  | -1.69 ;-0.49 | 0.12 ;0.73 | -0.02 ;0.74 |  |  |  |  |
|  |  | **Hierarchical Partitioning (%)** |  |  |  | 50.46 | 42.02 | 7.52 |  |  |  |  |
|  | **5**  **00** | **Estimate (±SE)** | -5.75(0.24) |  |  |  | 0.62(0.2) |  |  |  |  |  |
|  |  | **95% Confidence interval** | -6.22 ;-5.27 |  |  |  | 0.24 ;1 |  |  |  |  |  |
|  |  | **Hierarchical Partitioning (%)** |  |  |  |  | 100 |  |  |  |  |  |
|  | **750** | **Estimate (±SE)** | -5.81(0.3) |  |  |  | 0.66(0.22) |  | -1.68(0.58) | 1.27(0.48) | 0.72(0.23) | -0.61(0.35) |
|  |  | **95% Confidence interval** | -6.39 ;-5.24 |  |  |  | 0.24 ;1.09 |  | -2.81 ;-0.55 | 0.34 ;2.21 | 0.27 ;1.18 | -1.29 ; 0.07 |
|  |  | **Hierarchical Partitioning (%)** |  |  |  |  | 41.23 |  | 24.77 | 11.24 | 17.98 | 4.82 |
|  | **1000** | **Estimate (±SE)** | -5.8(0.25) |  |  |  | 0.72(0.23) |  |  |  |  |  |
|  |  | **95% Confidence interval** | -6.29 ;-5.31 |  |  |  | 0.26 ;1.17 |  |  |  |  |  |
|  |  | **Hierarchical Partitioning (%)** |  |  |  |  | 100 |  |  |  |  |  |
|  | **1500** | **Estimate (±SE)** | -5.78(0.3) |  | 1990.95(779.51) | 158.14(144.68) | 107.85(152.07) | 1788.98(700.55) |  | 0.42(0.33) | 0.49(0.27) |  |
|  |  | **95% Confidence interval** | -6.36 ;-5.2 |  | 463.14 ;3518.77 | -125.43 ;441.71 | -190.21 ;405.91 | 415.94 ;3162.03 |  | -0.23 ;1.06 | -0.05 ;1.03 |  |
|  |  | **Hierarchical Partitioning (%)** |  |  | 17.99 | 11.25 | 17.47 | 21.30 |  | 4.63 | 27.35 |  |
| *Trachops cirrhosus* | | | | **Local-scale** | **Compositional Predictors** | | | | **Configurational Predictors** | | | |
| **Season** | **Spatial Scale (m)** | **Explanatory Variables** | **Intercept** | **Vegetation**  **structure** | **FC** | **SF 1** | **SF 2** | **SF 3** | **PD** | **ED** | **MNND** | **MSI** |
| **Dry Season** | **250** | **Estimate (±SE)** | -6.05(0.41) | 0.54(0.37) |  | -0.78(0.39) |  |  |  |  | -0.36(0.32) |  |
|  |  | **95% Confidence interval** | -6.85 ;-5.25 | -0.18 ;1.26 |  | -1.54 ;-0.02 |  |  |  |  | -0.99 ;0.27 |  |
|  |  | **Hierarchical Partitioning (%)** |  | 57.57 |  | 16.89 |  |  |  |  | 25.54 |  |
|  | **500** | **Estimate (±SE)** | -5.87(0.49) | 0.54(0.37) |  |  |  | -0.35(0.38) |  | 0.56(0.31) | -0.36(0.33) | 0.35(0.25) |
|  |  | **95% Confidence interval** | -6.83 ;-4.91 | -0.18 ;1.26 |  |  |  | -1.09 ;0.38 |  | -0.05 ;1.17 | -1 ;0.28 | -0.14 ; 0.84 |
|  |  | **Hierarchical Partitioning (%)** |  | 36.27 |  |  |  | 24.56 |  | 10.10 | 25.68 | 3.39 |
|  | **750** | **Estimate (±SE)** | -5.72(0.38) |  |  |  |  |  |  |  |  | 0.62(0.15) |
|  |  | **95% Confidence interval** | -6.47 ;-4.97 |  |  |  |  |  |  |  |  | 0.33 ; 0.91 |
|  |  | **Hierarchical Partitioning (%)** |  |  |  |  |  |  |  |  |  | 100 |
|  | **1000** | **Estimate (±SE)** | -5.97(0.41) | 0.54(0.37) | 0.53(0.25) |  |  | -0.6(0.26) |  |  |  | 0.46(0.23) |
|  |  | **95% Confidence interval** | -6.77 ;-5.18 | -0.18 ;1.26 | 0.04 ;1.01 |  |  | -1.1 ;-0.09 |  |  |  | 0.01 ; 0.9 |
|  |  | **Hierarchical Partitioning (%)** |  | 34.73 | 27.02 |  |  | 23.63 |  |  |  | 14.63 |
|  | **1500** | **Estimate (±SE)** | -5.93(0.42) |  | 0.61(0.25) |  |  | -0.61(0.26) |  |  |  | 0.56(0.22) |
|  |  | **95% Confidence interval** | -6.75 ;-5.11 |  | 0.12 ;1.09 |  |  | -1.11 ;-0.11 |  |  |  | 0.13 ; 1 |
|  |  | **Hierarchical Partitioning (%)** |  |  | 38.63 |  |  | 30.81 |  |  |  | 30.56 |
| **Wet Season** | **250** | **Estimate (±SE)** | -5.35 (0.28) |  | 0.47(0.19) |  |  | -0.51(0.19) |  |  |  |  |
|  |  | **95% Confidence interval** | -5.89 ;-4.81 |  | 0.1 ;0.84 |  |  | -0.88 ;-0.13 |  |  |  |  |
|  |  | **Hierarchical Partitioning (%)** |  |  | 49.24 |  |  | 50.76 |  |  |  |  |
|  | **500** | **Estimate (±SE)** | -5.35(0.24) |  |  |  |  |  |  |  | -0.65(0.19) |  |
|  |  | **95% Confidence interval** | -5.83 ;-4.87 |  |  |  |  |  |  |  | -1.02 ;-0.29 |  |
|  |  | **Hierarchical Partitioning (%)** |  |  |  |  |  |  |  |  | 100 |  |
|  | **750** | **Estimate (±SE)** | -5.35(0.25) |  |  |  |  |  |  |  | -0.65(0.19) |  |
|  |  | **95% Confidence interval** | -5.84 ;-4.86 |  |  |  |  |  |  |  | -1.02 ;-0.28 |  |
|  |  | **Hierarchical Partitioning (%)** |  |  |  |  |  |  |  |  | 100 |  |
|  | **1000** | **Estimate (±SE)** | -5.37(0.25) |  |  |  |  |  |  |  | -0.66(0.18) |  |
|  |  | **95% Confidence interval** | -5.86 ;-4.88 |  |  |  |  |  |  |  | -1.02 ;-0.31 |  |
|  |  | **Hierarchical Partitioning (%)** |  |  |  |  |  |  |  |  | 100 |  |
|  | **1500** | **Estimate (±SE)** | -5.28(0.19) |  |  |  |  |  |  | -0.7(0.16) |  |  |
|  |  | **95% Confidence interval** | -5.65 ;-4.91 |  |  |  |  |  |  | -1.01 ;-0.39 |  |  |
|  |  | **Hierarchical Partitioning (%)** |  |  |  |  |  |  |  | 100 |  |  |
| *Pteronotus parnellii* | | | | **Local-scale** | **Compositional Predictors** | | | | **Configurational Predictors** | | | |
| **Season** | **Spatial Scale (m)** | **Explanatory Variables** | **Intercept** | **Vegetation**  **structure** | **FC** | **SF 1** | **SF 2** | **SF 3** | **PD** | **ED** | **MNND** | **MSI** |
| **Dry Season** | **250** | **Estimate (±SE)** | -4.56(0.24) |  |  | -0.45(0.15) |  |  |  |  |  |  |
|  |  | **95% Confidence interval** | -5.02 ;-4.09 |  |  | -0.74 ;-0.16 |  |  |  |  |  |  |
|  |  | **Hierarchical Partitioning (%)** |  |  |  | 100 |  |  |  |  |  |  |
|  | **500** | **Estimate (±SE)** | -4.59(0.25) |  | 0.37(0.13) | -0.46(0.16) |  | -0.36(0.13) |  |  | -0.37(0.15) |  |
|  |  | **95% Confidence interval** | -5.09 ;-4.09 |  | 0.11 ;0.64 | -0.78 ;-0.13 |  | -0.61 ;-0.11 |  |  | -0.66 ;-0.08 |  |
|  |  | **Hierarchical Partitioning (%)** |  |  | 31.18 | 13.03 |  | 27.67 |  |  | 28.13 |  |
|  | **750** | **Estimate (±SE)** | -4.49(0.19) |  |  | -0.47(0.18) |  |  |  |  |  |  |
|  |  | **95% Confidence interval** | -4.86 ;-4.13 |  |  | -0.83 ;-0.12 |  |  |  |  |  |  |
|  |  | **Hierarchical Partitioning (%)** |  |  |  | 100 |  |  |  |  |  |  |
|  | **1000** | **Estimate (±SE)** | -4.49(0.2) | 0.19(0.15) |  | -0.46(0.22) | 0.3(0.14) | -0.09(0.15) |  |  | -0.21(0.14) |  |
|  |  | **95% Confidence interval** | -4.89 ;-4.09 | -0.09 ;0.48 |  | -0.88 ;-0.03 | 0.04 ;0.57 | -0.37 ;0.2 |  |  | -0.48 ;0.06 |  |
|  |  | **Hierarchical Partitioning (%)** |  | 26.39 |  | 14.21 | 11.22 | 9.60 |  |  | 38.58 |  |
|  | **1500** | **Estimate (±SE)** | -4.6(0.26) |  |  |  |  |  |  |  | -0.31(0.14) |  |
|  |  | **95% Confidence interval** | -5.11 ;-4.09 |  |  |  |  |  |  |  | -0.58 ;-0.03 |  |
|  |  | **Hierarchical Partitioning (%)** |  |  |  |  |  |  |  |  | 100 |  |
| **Wet Season** | **250** | **Estimate (±SE)** | -4.5( 0.14) |  |  |  |  |  | 0.55(0.21) | -0.81(0.2) | -0.28(0.15) | 0.41(0.19) |
|  |  | **95% Confidence interval** | -4.77 ;-4.23 |  |  |  |  |  | 0.14 ;0.96 | -1.21 ;-0.41 | -0.58 ;0.02 | 0.04 ; 0.77 |
|  |  | **Hierarchical Partitioning (%)** |  |  |  |  |  |  | 11.06 | 49.35 | 16.77 | 22.82 |
|  | **500** | **Estimate (±SE)** | -4.5(0.15) |  | 0.4(0.13) |  |  | -0.4(0.13) |  |  |  |  |
|  |  | **95% Confidence interval** | -4.79 ;-4.21 |  | 0.15 ;0.66 |  |  | -0.66 ;-0.14 |  |  |  |  |
|  |  | **Hierarchical Partitioning (%)** |  |  | 53.25 |  |  | 46.75 |  |  |  |  |
|  | **750** | **Estimate (±SE)** | -4.5(0.15) |  | 0.4(0.13) |  |  | -0.4(0.13) |  |  |  |  |
|  |  | **95% Confidence interval** | -4.79 ;-4.21 |  | 0.15 ;0.65 |  |  | -0.65 ;-0.14 |  |  |  |  |
|  |  | **Hierarchical Partitioning (%)** |  |  | 54.20 |  |  | 45.80 |  |  |  |  |
|  | **1000** | **Estimate (±SE)** | -4.49(0.15) |  | 0.38(0.13) |  |  | -0.38(0.13) |  |  |  |  |
|  |  | **95% Confidence interval** | -4.78 ;-4.21 |  | 0.13 ;0.64 |  |  | -0.64 ;-0.12 |  |  |  |  |
|  |  | **Hierarchical Partitioning (%)** |  |  | 54.70 |  |  | 45.30 |  |  |  |  |
|  | **1500** | **Estimate (±SE)** | -4.49(0.15) |  | 0.38(0.13) |  |  | -0.37(0.13) |  |  | -0.35(0.13) |  |
|  |  | **95% Confidence interval** | -4.78 ;-4.2 |  | 0.12 ;0.63 |  |  | -0.62 ;-0.11 |  |  | -0.61 ;-0.1 |  |
|  |  | **Hierarchical Partitioning (%)** |  |  | 33.73 |  |  | 28.08 |  |  | 38.19 |  |

**Table S9** Best-fit models (∆AIC_c_ ≤ 2) investigating the relationship between local and landscape-scale attributes and abundance of eight species for the wet and dry seasons and for five focal scales across the BDFFP. Central Amazon. Brazil. For each model. the number of estimated parameters (*K*). sample-size adjusted Akaike’s information criterion (AIC_c_). Akaike differences (Δ_i_). Akaike weights (*w*_i_). cumulative Akaike weight (Cum*_W_*) and log-likelihood (log(L)) are presented. Predictor abbreviations: LVS – local vegetation structure; PFC – primary forest cover; SFC1 – initial secondary forest cover; SFC2 – intermediate secondary forest cover; SFC3 – advanced secondary forest cover; ED – edge density; PD – patch density; MNND – mean nearest neighbour distance; MSI – mean shape index.

| *Artibeus obscurus* | | | | | | | | |
| --- | --- | --- | --- | --- | --- | --- | --- | --- |
| **Season** | **Spatial Scale (m)** | **Model structure** | ***K*** | **AIC_c_** | **Δ_i_** | ***w*_i_** | **Cum*_w_*** | **log(L)** |
| **Dry Season** | **250** | SFC1 | 4 | 137.20 | 0 | 0.28 | 0.28 | -64.01 |
|  |  | LVS+SFC1+SFC2+SFC3+ED+PD+MNND+MSI | 11 | 138.98 | 1.78 | 0.12 | 0.40 | -53.60 |
|  |  | ED | 4 | 139.01 | 1.81 | 0.11 | 0.51 | -64.92 |
|  |  | PD | 4 | 139.09 | 1.89 | 0.11 | 0.62 | -64.96 |
|  | **500** | SFC3 | 4 | 139.14 | 0 | 0.20 | 0.20 | -64.98 |
|  |  | LVS | 4 | 139.39 | 0.25 | 0.17 | 0.37 | -65.11 |
|  |  | PFC | 4 | 139.41 | 0.27 | 0.17 | 0.55 | -65.12 |
|  |  | SFC1 | 4 | 140.91 | 1.76 | 0.08 | 0.63 | -65.87 |
|  | **750** | SFC3 | 4 | 138.10 | 0 | 0.30 | 0.30 | -64.46 |
|  |  | PFC | 4 | 139.32 | 1.22 | 0.16 | 0.46 | -65.07 |
|  |  | LVS | 4 | 139.39 | 1.29 | 0.16 | 0.62 | -65.11 |
|  | **1000** | SFC3 | 4 | 138.19 | 0 | 0.22 | 0.22 | -64.51 |
|  |  | SFC1+SFC2+SFC3 | 6 | 138.84 | 0.65 | 0.16 | 0.38 | -62.11 |
|  |  | LVS | 4 | 139.39 | 1.20 | 0.12 | 0.50 | -65.11 |
|  |  | PD | 4 | 139.53 | 1.34 | 0.11 | 0.61 | -65.17 |
|  |  | PFC | 4 | 139.80 | 1.61 | 0.10 | 0.71 | -65.31 |
|  | **1500** | SFC1+SFC2+SFC3 | 6 | 132.84 | 0 | 0.52 | 0.52 | -59.11 |
| **Wet Season** | **250** | SFC2 | 4 | 100.74 | 0 | 0.24 | 0.24 | -45.78 |
|  |  | LVS | 4 | 101.21 | 0.46 | 0.19 | 0.43 | -46.02 |
|  |  | PFC | 4 | 101.31 | 0.57 | 0.18 | 0.61 | -46.07 |
|  |  | SFC1 | 4 | 102.06 | 1.32 | 0.12 | 0.73 | -46.44 |
|  |  | SFC3 | 4 | 102.73 | 1.99 | 0.09 | 0.82 | -46.78 |
|  | **500** | PD | 4 | 100.12 | 0 | 0.29 | 0.29 | -45.47 |
|  |  | LVS | 4 | 101.21 | 1.09 | 0.17 | 0.46 | -46.02 |
|  |  | SFC3 | 4 | 101.87 | 1.75 | 0.12 | 0.59 | -46.35 |
|  |  | PFC | 4 | 102.03 | 1.91 | 0.11 | 0.70 | -46.43 |
|  | **750** | LVS | 4 | 101.21 | 0 | 0.28 | 0.28 | -46.02 |
|  |  | MNND | 4 | 102.86 | 1.66 | 0.12 | 0.40 | -46.84 |
|  |  | PFC | 4 | 103.10 | 1.89 | 0.11 | 0.51 | -46.96 |
|  |  | SFC3 | 4 | 103.17 | 1.96 | 0.10 | 0.61 | -47 |
|  | **1000** | LVS | 4 | 101.21 | 0 | 0.33 | 0.33 | -46.02 |
|  |  | MNND | 4 | 102.92 | 1.71 | 0.14 | 0.47 | -46.87 |
|  | **1500** | SFC2 | 4 | 101.10 | 0 | 0.25 | 0.25 | -45.96 |
|  |  | LVS | 4 | 101.21 | 0.10 | 0.23 | 0.48 | -46.02 |
|  |  | MNND | 4 | 102.55 | 1.45 | 0.12 | 0.60 | -46.69 |
| *Carollia brevicauda* | | | | | | | | |
| **Season** | **Spatial Scale (m)** | **Model structure** | ***K*** | **AIC_c_** | **Δ_i_** | ***w*_i_** | **Cum*_w_*** | **log(L)** |
| **Dry Season** | **250** | PFC | 4 | 99.32 | 0 | 0.24 | 0.24 | -45.07 |
|  |  | LVS | 4 | 100.15 | 0.83 | 0.16 | 0.40 | -45.49 |
|  |  | SFC3 | 4 | 100.60 | 1.27 | 0.13 | 0.53 | -45.71 |
|  |  | LVS+ED+PD+MNND+MSI | 8 | 100.85 | 1.53 | 0.11 | 0.65 | -40.03 |
|  |  | ED | 4 | 101.19 | 1.87 | 0.10 | 0.74 | -46.01 |
|  | **500** | LVS | 4 | 100.15 | 0 | 0.31 | 0.31 | -45.49 |
|  |  | PFC | 4 | 100.57 | 0.42 | 0.25 | 0.56 | -45.70 |
|  |  | SFC3 | 4 | 101.04 | 0.89 | 0.20 | 0.76 | -45.93 |
|  | **750** | LVS | 4 | 100.15 | 0 | 0.32 | 0.32 | -45.49 |
|  |  | SFC3 | 4 | 100.80 | 0.65 | 0.23 | 0.55 | -45.81 |
|  |  | PFC | 4 | 101.71 | 1.55 | 0.15 | 0.70 | -46.27 |
|  | **1000** | LVS | 4 | 100.15 | 0 | 0.24 | 0.24 | -45.49 |
|  |  | LVS+PFC+ED+PD+MNND+MSI | 9 | 100.53 | 0.38 | 0.20 | 0.44 | -38.16 |
|  |  | SFC3 | 4 | 101.48 | 1.33 | 0.12 | 0.56 | -46.15 |
|  | **1500** | ED+PD+MNND+MSI | 7 | 90.59 | 0 | 0.47 | 0.47 | -36.49 |
|  |  | LVS+ED+PD+MNND+MSI | 8 | 90.79 | 0.20 | 0.42 | 0.89 | -35 |
| **Wet Season** | **250** | SFC3 | 4 | 165.50 | 0 | 0.39 | 0.39 | -78.16 |
|  |  | LVS | 4 | 166.47 | 0.96 | 0.24 | 0.63 | -78.65 |
|  |  | PFC | 4 | 167.24 | 1.74 | 0.16 | 0.80 | -79.03 |
|  | **500** | LVS | 4 | 166.47 | 0 | 0.23 | 0.23 | -78.65 |
|  |  | SFC3 | 4 | 166.96 | 0.49 | 0.18 | 0.40 | -78.89 |
|  |  | PFC | 4 | 167.61 | 1.14 | 0.13 | 0.53 | -79.22 |
|  |  | MSI | 4 | 168.22 | 1.76 | 0.09 | 0.63 | -79.52 |
|  | **750** | LVS+PFC+ED+PD+MNND+MSI | 9 | 165.51 | 0 | 0.32 | 0.32 | -70.65 |
|  |  | LVS | 4 | 166.47 | 0.96 | 0.20 | 0.51 | -78.65 |
|  |  | LVS+ED+PD+MNND+MSI | 8 | 167.28 | 1.78 | 0.13 | 0.64 | -73.24 |
|  | **1000** | LVS | 4 | 166.47 | 0 | 0.50 | 0.50 | -78.65 |
|  | **1500** | MNND | 4 | 165.35 | 0 | 0.46 | 0.46 | -78.09 |
|  |  | LVS | 4 | 166.47 | 1.12 | 0.27 | 0.73 | -78.65 |
| *Carollia perspicillata* | | | | | | | | |
| **Season** | **Spatial Scale (m)** | **Model structure** | ***K*** | **AIC_c_** | **Δ_i_** | ***w*_i_** | **Cum*_w_*** | **log(L)** |
| **Dry Season** | **250** | LVS+ED+PD+MNND+MSI | 8 | 317.37 | 0 | 0.40 | 0.40 | -148.28 |
|  |  | LVS+PFC+ED+PD+MNND+MSI | 9 | 318.51 | 1.14 | 0.23 | 0.63 | -147.15 |
|  |  | PFC | 4 | 319.06 | 1.70 | 0.17 | 0.80 | -154.94 |
|  | **500** | PFC | 4 | 315.94 | 0 | 0.36 | 0.36 | -153.38 |
|  |  | LVS+PFC+ED+PD+MNND+MSI | 9 | 316.69 | 0.75 | 0.25 | 0.62 | -146.24 |
|  |  | SFC3 | 4 | 317.70 | 1.75 | 0.15 | 0.77 | -154.26 |
|  | **750** | SFC3 | 4 | 313.29 | 0 | 0.56 | 0.56 | -152.06 |
|  |  | PFC | 4 | 315.24 | 1.95 | 0.21 | 0.77 | -153.03 |
|  | **1000** | SFC3 | 4 | 314.21 | 0 | 0.51 | 0.51 | -152.51 |
|  |  | LVS+PFC+ED+PD+MNND+MSI | 9 | 315.91 | 1.71 | 0.22 | 0.72 | -145.85 |
|  | **1500** | LVS+PFC+ED+PD+MNND+MSI | 9 | 312.48 | 0 | 0.45 | 0.45 | -144.14 |
| **Wet Season** | **250** | LVS+ED+PD+MNND+MSI | 8 | 335.62 | 0 | 0.48 | 0.48 | -157.41 |
|  | **500** | LVS+PFC+ED+PD+MNND+MSI | 9 | 325.26 | 0 | 0.89 | 0.89 | -150.53 |
|  | **750** | LVS+PFC+ED+PD+MNND+MSI | 9 | 326.73 | 0 | 0.90 | 0.90 | -151.26 |
|  | **1000** | PFC | 4 | 333.67 | 0 | 0.38 | 0.38 | -162.25 |
|  |  | SFC3 | 4 | 334.06 | 0.39 | 0.31 | 0.70 | -162.44 |
|  |  | LVS+PFC+ED+PD+MNND+MSI | 9 | 334.98 | 1.31 | 0.20 | 0.90 | -155.39 |
|  | **1500** | SFC3 | 4 | 332.67 | 0 | 0.52 | 0.52 | -161.75 |
| *Rhinophylla pumilio* | | | | | | | | |
| **Season** | **Spatial Scale (m)** | **Model structure** | ***K*** | **AIC_c_** | **Δ_i_** | ***w*_i_** | **Cum*_w_*** | **log(L)** |
| **Dry Season** | **250** | SFC1 | 4 | 193.29 | 0 | 0.32 | 0.32 | -92.06 |
|  |  | ED | 4 | 193.97 | 0.69 | 0.23 | 0.55 | -92.40 |
|  | **500** | PD | 4 | 197.37 | 0 | 0.14 | 0.14 | -94.10 |
|  |  | SFC2 | 4 | 197.67 | 0.30 | 0.12 | 0.26 | -94.25 |
|  |  | ED | 4 | 197.69 | 0.32 | 0.12 | 0.38 | -94.26 |
|  |  | PFC | 4 | 198.19 | 0.82 | 0.09 | 0.47 | -94.51 |
|  |  | SFC3 | 4 | 198.21 | 0.84 | 0.09 | 0.56 | -94.52 |
|  |  | LVS | 4 | 198.22 | 0.85 | 0.09 | 0.65 | -94.52 |
|  |  | MSI | 4 | 198.30 | 0.93 | 0.09 | 0.74 | -94.56 |
|  |  | SFC1 | 4 | 198.32 | 0.95 | 0.09 | 0.82 | -94.57 |
|  |  | MNND | 4 | 198.41 | 1.04 | 0.08 | 0.91 | -94.62 |
|  | **750** | LVS+PFC+ED+PD+MNND+MSI | 9 | 195.21 | 0 | 0.26 | 0.26 | -85.50 |
|  |  | PD | 4 | 197.11 | 1.90 | 0.10 | 0.35 | -93.97 |
|  | **1000** | SFC3 | 4 | 196.71 | 0 | 0.17 | 0.17 | -93.77 |
|  |  | PFC | 4 | 196.83 | 0.12 | 0.16 | 0.32 | -93.83 |
|  |  | MSI | 4 | 197.52 | 0.81 | 0.11 | 0.43 | -94.17 |
|  |  | PD | 4 | 197.68 | 0.97 | 0.10 | 0.53 | -94.25 |
|  |  | SFC1 | 4 | 197.94 | 1.24 | 0.09 | 0.62 | -94.38 |
|  |  | ED | 4 | 197.98 | 1.28 | 0.09 | 0.71 | -94.40 |
|  |  | MNND | 4 | 198.17 | 1.46 | 0.08 | 0.79 | -94.50 |
|  |  | SFC2 | 4 | 198.21 | 1.50 | 0.08 | 0.87 | -94.52 |
|  |  | LVS | 4 | 198.22 | 1.51 | 0.08 | 0.94 | -94.52 |
|  | **1500** | SFC3 | 4 | 196.05 | 0 | 0.19 | 0.19 | -93.44 |
|  |  | PFC | 4 | 196.23 | 0.18 | 0.18 | 0.37 | -93.53 |
|  |  | SFC1 | 4 | 196.82 | 0.76 | 0.13 | 0.50 | -93.82 |
|  |  | MNND | 4 | 197.07 | 1.02 | 0.12 | 0.62 | -93.95 |
| **Wet Season** | **250** | SFC3 | 4 | 219.36 | 0 | 0.27 | 0.27 | -105.09 |
|  |  | PFC | 4 | 219.93 | 0.58 | 0.20 | 0.47 | -105.38 |
|  |  | LVS | 4 | 220.78 | 1.42 | 0.13 | 0.60 | -105.80 |
|  |  | SFC1+SFC2+SFC3 | 6 | 220.98 | 1.62 | 0.12 | 0.72 | -103.18 |
|  | **500** | SFC2 | 4 | 219.34 | 0 | 0.24 | 0.24 | -105.08 |
|  |  | PFC | 4 | 219.73 | 0.39 | 0.20 | 0.44 | -105.28 |
|  |  | ED | 4 | 220.33 | 1 | 0.15 | 0.58 | -105.58 |
|  |  | LVS | 4 | 220.78 | 1.45 | 0.12 | 0.70 | -105.80 |
|  | **750** | PFC | 4 | 218.15 | 0 | 0.42 | 0.42 | -104.49 |
|  | **1000** | PFC | 4 | 217.62 | 0 | 0.40 | 0.40 | -104.22 |
|  |  | MNND | 4 | 218.68 | 1.06 | 0.23 | 0.63 | -104.75 |
|  | **1500** | MNND | 4 | 218.41 | 0 | 0.31 | 0.31 | -104.61 |
|  |  | PFC | 4 | 218.81 | 0.40 | 0.25 | 0.56 | -104.82 |
|  |  | SFC3 | 4 | 220.23 | 1.82 | 0.12 | 0.69 | -105.53 |
| *Lophostoma silvicolum* | | | | | | | | |
| **Season** | **Spatial Scale (m)** | **Model structure** | ***K*** | **AIC_c_** | **Δ_i_** | ***w*_i_** | **Cum*_w_*** | **log(L)** |
| **Dry Season** | **250** | LVS | 4 | 93.09 | 0 | 0.22 | 0.22 | -41.96 |
|  |  | SFC3 | 4 | 93.69 | 0.60 | 0.16 | 0.38 | -42.26 |
|  |  | MNND | 4 | 94.49 | 1.39 | 0.11 | 0.49 | -42.65 |
|  |  | PFC | 4 | 94.62 | 1.53 | 0.10 | 0.59 | -42.72 |
|  |  | ED | 4 | 94.82 | 1.73 | 0.09 | 0.69 | -42.82 |
|  | **500** | ED+PD+MNND+MSI | 7 | 87.20 | 0 | 0.45 | 0.45 | -34.79 |
|  |  | PD | 4 | 88.84 | 1.64 | 0.20 | 0.64 | -39.83 |
|  | **750** | PD | 4 | 86.46 | 0 | 0.64 | 0.64 | -38.64 |
|  | **1000** | PD | 4 | 82.29 | 0 | 0.82 | 0.82 | -36.56 |
|  | **1500** | PD | 4 | 84.55 | 0 | 0.68 | 0.68 | -37.69 |
| **Wet Season** | **250** | SFC1+SFC2+SFC3 | 6 | 123.40 | 0 | 0.21 | 0.21 | -54.39 |
|  |  | SFC2 | 4 | 123.93 | 0.53 | 0.16 | 0.37 | -57.38 |
|  |  | SFC3 | 4 | 124 | 0.60 | 0.15 | 0.52 | -57.41 |
|  |  | PFC | 4 | 124.50 | 1.10 | 0.12 | 0.64 | -57.66 |
|  |  | LVS | 4 | 124.80 | 1.40 | 0.10 | 0.75 | -57.81 |
|  |  | PFC+SFC1+SFC2+SFC3 | 7 | 125.25 | 1.85 | 0.08 | 0.83 | -53.82 |
|  | **500** | MNND | 4 | 116.74 | 0 | 0.64 | 0.64 | -53.78 |
|  | **750** | MNND | 4 | 115.28 | 0 | 0.62 | 0.62 | -53.05 |
|  | **1000** | PD | 4 | 117.35 | 0 | 0.43 | 0.43 | -54.09 |
|  |  | SFC2 | 4 | 117.45 | 0.10 | 0.40 | 0.83 | -54.14 |
|  | **1500** | PD | 4 | 119.69 | 0 | 0.40 | 0.40 | -55.26 |
|  |  | ED | 4 | 120.58 | 0.89 | 0.26 | 0.65 | -55.70 |
|  |  | SFC2 | 4 | 121.10 | 1.41 | 0.20 | 0.85 | -55.96 |
| *Mimon crenulatum* | | | | | | | | |
| **Season** | **Spatial Scale (m)** | **Model structure** | ***K*** | **AIC_c_** | **Δ_i_** | ***w*_i_** | **Cum*_w_*** | **log(L)** |
| **Dry Season** | **250** | SFC1 | 4 | 104.57 | 0 | 0.51 | 0.51 | -47.70 |
|  | **500** | SFC3 | 4 | 107.33 | 0 | 0.19 | 0.19 | -49.08 |
|  |  | SFC1 + SFC2 + SFC3 | 6 | 107.57 | 0.24 | 0.17 | 0.36 | -46.47 |
|  |  | MNND | 4 | 107.59 | 0.26 | 0.17 | 0.53 | -49.21 |
|  |  | SFC1 | 4 | 108.37 | 1.04 | 0.11 | 0.65 | -49.60 |
|  |  | PFC | 4 | 108.42 | 1.09 | 0.11 | 0.76 | -49.62 |
|  |  | PD | 4 | 109.14 | 1.81 | 0.08 | 0.84 | -49.98 |
|  | **750** | SFC3 | 4 | 107.77 | 0 | 0.29 | 0.29 | -49.30 |
|  |  | PFC | 4 | 108.48 | 0.71 | 0.20 | 0.50 | -49.65 |
|  | **1000** | SFC3 | 4 | 108.08 | 0 | 0.26 | 0.26 | -49.45 |
|  |  | PFC | 4 | 108.80 | 0.71 | 0.18 | 0.44 | -49.81 |
|  | **1500** | SFC3 | 4 | 107.51 | 0 | 0.26 | 0.26 | -49.17 |
|  |  | PFC | 4 | 108.02 | 0.51 | 0.20 | 0.46 | -49.42 |
| **Wet Season** | **250** | SFC1+SFC2+SFC3 | 6 | 116.11 | 0 | 0.61 | 0.61 | -50.74 |
|  | **500** | SFC2 | 4 | 124.06 | 0 | 0.56 | 0.56 | -57.44 |
|  | **750** | SFC2 | 4 | 123.92 | 0 | 0.42 | 0.42 | -57.37 |
|  |  | ED+PD+MNND+MSI | 7 | 124.99 | 1.07 | 0.25 | 0.67 | -53.69 |
|  | **1000** | SFC2 | 4 | 123.62 | 0 | 0.58 | 0.58 | -57.22 |
|  | **1500** | SFC2 | 4 | 129.33 | 0 | 0.22 | 0.22 | -60.08 |
|  |  | MNND | 4 | 129.99 | 0.66 | 0.16 | 0.38 | -60.40 |
|  |  | PFC+SFC1+SFC2+SFC3 | 7 | 130.28 | 0.95 | 0.14 | 0.52 | -56.33 |
|  |  | ED | 4 | 130.57 | 1.24 | 0.12 | 0.64 | -60.70 |
|  |  | SFC1 | 4 | 131.31 | 1.98 | 0.08 | 0.72 | -61.07 |
| *Trachops cirrhosus* | | | | | | | | |
| **Season** | **Spatial Scale (m)** | **Model structure** | ***K*** | **AIC_c_** | **Δ_i_** | ***w*_i_** | **Cum*_w_*** | **log(L)** |
| **Dry Season** | **250** | SFC1 | 4 | 101.15 | 0 | 0.28 | 0.28 | -45.99 |
|  |  | LVS | 4 | 101.94 | 0.79 | 0.19 | 0.48 | -46.38 |
|  |  | MNND | 4 | 103.13 | 1.98 | 0.11 | 0.58 | -46.98 |
|  | **500** | ED | 4 | 101.68 | 0 | 0.21 | 0.21 | -46.25 |
|  |  | LVS | 4 | 101.94 | 0.25 | 0.18 | 0.39 | -46.38 |
|  |  | MSI | 4 | 102.55 | 0.87 | 0.13 | 0.52 | -46.69 |
|  |  | MNND | 4 | 103.30 | 1.62 | 0.09 | 0.62 | -47.06 |
|  |  | SFC3 | 4 | 103.62 | 1.94 | 0.08 | 0.69 | -47.22 |
|  | **750** | MSI | 4 | 94.89 | 0 | 0.71 | 0.71 | -42.86 |
|  | **1000** | SFC3 | 4 | 100.75 | 0 | 0.22 | 0.22 | -45.79 |
|  |  | MSI | 4 | 100.94 | 0.19 | 0.20 | 0.42 | -45.88 |
|  |  | PFC | 4 | 101.60 | 0.86 | 0.14 | 0.57 | -46.21 |
|  |  | LVS | 4 | 101.94 | 1.19 | 0.12 | 0.69 | -46.38 |
|  | **1500** | MSI | 4 | 98.84 | 0 | 0.34 | 0.34 | -44.83 |
|  |  | PFC | 4 | 100.02 | 1.17 | 0.19 | 0.52 | -45.42 |
|  |  | SFC3 | 4 | 100.28 | 1.44 | 0.16 | 0.69 | -45.55 |
| **Wet Season** | **250** | SFC3 | 4 | 145.85 | 0 | 0.39 | 0.39 | -68.34 |
|  |  | PFC | 4 | 146.67 | 0.82 | 0.26 | 0.66 | -68.75 |
|  | **500** | MNND | 4 | 141.63 | 0 | 0.70 | 0.70 | -66.23 |
|  | **750** | MNND | 4 | 141.45 | 0 | 0.73 | 0.73 | -66.14 |
|  | **1000** | MNND | 4 | 139.86 | 0 | 0.79 | 0.79 | -65.34 |
|  | **1500** | ED | 4 | 139.89 | 0 | 0.68 | 0.68 | -65.36 |
| *Pteronotus parnellii* | | | | | | | | |
| **Season** | **Spatial Scale (m)** | **Model structure** | ***K*** | **AIC_c_** | **Δ_i_** | ***w*_i_** | **Cum*_w_*** | **log(L)** |
| **Dry Season** | **250** | SFC1 | 4 | 145.71 | 0 | 0.47 | 0.47 | -68.26 |
|  | **500** | PFC | 4 | 147.59 | 0 | 0.27 | 0.27 | -69.21 |
|  |  | SFC3 | 4 | 147.68 | 0.09 | 0.26 | 0.53 | -69.25 |
|  |  | SFC1 | 4 | 148.03 | 0.44 | 0.22 | 0.75 | -69.43 |
|  |  | MNND | 4 | 149.25 | 1.66 | 0.12 | 0.87 | -70.04 |
|  | **750** | SFC1 | 4 | 148.91 | 0 | 0.46 | 0.46 | -69.87 |
|  | **1000** | SFC1 | 4 | 151.50 | 0 | 0.22 | 0.22 | -71.16 |
|  |  | SFC1+SFC2+SFC3 | 6 | 152.22 | 0.72 | 0.15 | 0.37 | -68.80 |
|  |  | MNND | 4 | 152.85 | 1.34 | 0.11 | 0.48 | -71.83 |
|  |  | LVS | 4 | 153.42 | 1.92 | 0.08 | 0.56 | -72.12 |
|  | **1500** | MNND | 4 | 150.74 | 0 | 0.44 | 0.44 | -70.78 |
| **Wet Season** | **250** | ED+PD+MNND+MSI | 7 | 192.85 | 0 | 0.49 | 0.49 | -87.62 |
|  | **500** | PFC | 4 | 192.95 | 0 | 0.38 | 0.38 | -91.89 |
|  |  | SFC3 | 4 | 193.54 | 0.59 | 0.28 | 0.66 | -92.18 |
|  | **750** | PFC | 4 | 192.89 | 0 | 0.44 | 0.44 | -91.86 |
|  |  | SFC3 | 4 | 193.51 | 0.62 | 0.32 | 0.76 | -92.17 |
|  | **1000** | PFC | 4 | 193.69 | 0 | 0.44 | 0.44 | -92.26 |
|  |  | SFC3 | 4 | 194.27 | 0.58 | 0.33 | 0.76 | -92.55 |
|  | **1500** | PFC | 4 | 194.13 | 0 | 0.31 | 0.31 | -92.48 |
|  |  | SFC3 | 4 | 194.96 | 0.83 | 0.21 | 0.52 | -92.89 |
|  |  | MNND | 4 | 195.17 | 1.04 | 0.19 | 0.70 | -93 |
